# Combining Nickel- and Zinc-Porphyrin Sites via Covalent Organic Frameworks for Electrochemical CO_2_ Reduction

**DOI:** 10.1021/acsami.4c02511

**Published:** 2024-06-24

**Authors:** Hugo Veldhuizen, Maryam Abdinejad, Pieter J. Gilissen, Jelco Albertsma, Thomas Burdyny, Frans D. Tichelaar, Sybrand van der Zwaag, Monique A. van der Veen

**Affiliations:** a Novel Aerospace Materials, Faculty of Aerospace Engineering, Technische Universiteit Delft, 2629 HS Delft, The Netherlands; b Catalysis Engineering, Faculty of Applied Sciences, Technische Universiteit Delft, 2629 HZ Delft, The Netherlands; c Materials for Energy Conversion and Storage, Faculty of Applied Sciences, Technische Universiteit Delft, 2629 HZ Delft, The Netherlands; d Molecular Nanotechnology, Institute for Molecules and Materials, Radboud Universiteit, 6525 AJ Nijmegen, The Netherlands; e Kavli Institute of Nanoscience, Quantum Nanoscience, Physics Building, Technische Universiteit Delft, 2628 CJ Delft, The Netherlands

**Keywords:** covalent organic frameworks, Ni- and Zn-porphyrins, CO_2_ electroreduction, CO_2_RR, electrolysis, bifunctional catalysis

## Abstract

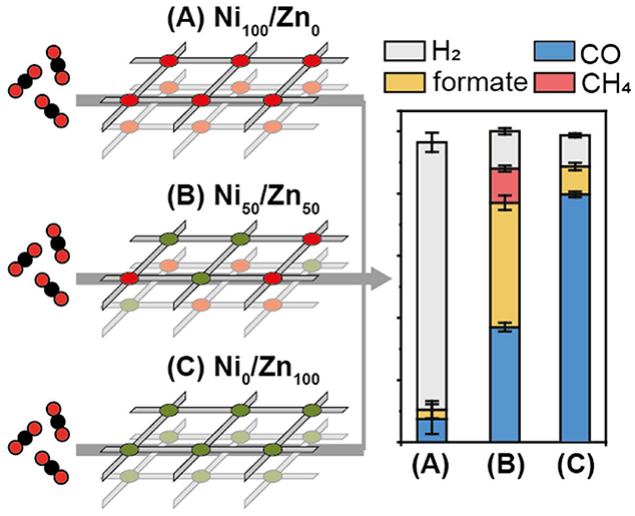

Covalent organic frameworks (COFs) are ideal platforms to spatially control the integration of multiple molecular motifs throughout a single nanoporous framework. Despite this design flexibility, COFs are typically synthesized using only two monomers. One bears the functional motif for the envisioned application, while the other is used as an inert connecting building block. Integrating more than one functional motif extends the functionality of COFs immensely, which is particularly useful for multistep reactions such as electrochemical reduction of CO_2_. In this systematic study, we synthesized five Ni(II)- and Zn(II)-porphyrin-based COFs, including two pure component COFs (Ni_100_ and Zn_100_) and three mixed Ni/Zn-COFs (Ni_75_/Zn_25_, Ni_50_/Zn_50_, and Ni_25_/Zn_75_). Among these, the Ni_50_/Zn_50_-COF exhibited the highest catalytic performance for the electroreduction of CO_2_ to CO and formate at −0.6 V vs RHE, as was observed in an H-cell. The catalytic performance of the COF catalysts was further extended to a zero-gap membrane electrode assembly (MEA) operation where, utilizing Ni_50_/Zn_50_, CH_4_ was detected along with CO and formate at a high current density of 150 mA cm^–2^. In contrast, under these conditions predominantly H_2_ and CO were detected at Ni_100_ and Zn_100_ respectively, indicating a clear synergistic effect between the Ni- and Zn-porphyrin units.

## 1 Introduction

Electrochemical carbon dioxide reduction reaction (CO_2_RR) into C_1_ products such as CO, formate, CH_4_, and methanol using renewable electrical energy is a promising route toward fossil-fuel-free feedstock.^1^ Since CO_2_ has a high reduction reaction energy barrier, catalysts are required for its conversion.^2^ Well-studied materials for this purpose are transition metal particles and surfaces,^3,4^ as well as molecular catalysts.^5^ Continuous optimization of these catalysts aims to improve their stability, efficiency, and product selectivity, while new catalytic designs offer promise for new functionalities. However, controlling the product species through easily implementable chemical modifications of the catalyst remains a challenge.

Covalent organic frameworks (COFs) and metal–organic frameworks (MOFs) are emerging alternative catalysts that have shown a promising approach toward electroreduction of CO_2_ with high tunability.^6^ Careful design of their originating monomers allows chemical and spatial control of their active sites, resulting in high degrees of control over product selectivity.^7,8^ At the same time, their nanoporous networks make the active sites highly accessible to CO_2_ and product intermediates. COFs with strong polymer backbones, such as “locked” polyimines and polyimides, are receiving more attention due to their decent stability in aqueous electrolytes.^9,10^ Widely used catalytically active units in these COFs are porphyrins and phthalocyanines.^11^ These molecular motifs are highly tunable, since the type of metal-ion coordinated to the porphyrin or phthalocyanine ligand greatly impacts specific electro- or photocatalytic reactions.^12,13^ For example, in porphyrin-based COFs, the optimal metal-ion in the production of CO during electrochemical CO_2_RR is cobalt.^14,15^ On the other hand, Ni- and Zn-porphyrin-based COFs outperform Co-porphyrin-COFs in the photocatalytic hydrogen evolution reaction (HER).^16^ Much of the catalytic activity of these materials depends on the electronic configuration of the coordination complex between the metal-ion and the porphyrin ligands. This presents opportunities for the development of bifunctional (A-B) catalysts, where regulation of the ratio of A to B may provide control over the activity and selectivity during CO_2_ electroreduction.

A bifunctional strategy has been employed in phthalocyanine MOF systems, where control of both the metal node of the MOF backbone and the metal ion coordinated to the phthalocyanine allows the formation of a true A-B bifunctional catalyst. For example, Zhong et al.^17^ studied a system using either copper- or zinc-phthalocyanine linkers in MOFs structured by either copper- or zinc-bis(dihydroxy) nodes, totaling 4 different structures. Interestingly, the specific combination of a copper-phthalocyanine combined with a ZnO_4_ backbone produced the largest Faradaic efficiency (FE) of 88% CO and a current density (*j*) of 4 mA cm^–2^ at −0.7 V vs a reversible hydrogen electrode (RHE). The conversions on the bifunctional catalyst were attributed to transfer of protons and electrons that were initially generated on the copper-phthalocyanine sites during water electrolysis and were then transported to the zinc-active sites where CO_2_ could be efficiently converted into CO. In a similar system using copper-phthalocyanine linkers and CuO_4_ nodes, Chen and co-workers^18^ demonstrated that C_2_H_4_ can be produced with FE = 50% and *j* = 7.3 mA cm^–2^ at −1.2 V vs RHE. They proposed that CO formation, followed by desorption from the copper-node of the backbone and finally diffusion toward the CO-copper-phthalocyanine active site, enables C–C coupling. Thus, the incorporation of multiple catalytically active units within one framework may provide cooperativity in terms of the reaction mechanism.

In this work, we employed a systematic approach of mixing nickel- and zinc-porphyrin monomers at various ratios. Each monomer mixture was then utilized in the COF polymerization process, yielding multiple different COF catalysts for CO_2_RR. The COFs were synthesized under solvothermal conditions with tetraamino-functionalized metalloporphyrins with terephthaldehyde linkers. The molar ratios of nickel- to zinc-porphyrin units were varied: Ni_100_/Zn_0_, Ni_75_/Zn_25_, Ni_50_/Zn_50_, Ni_25_/Zn_75_, and Ni_0_/Zn_100_, totaling five different frameworks. After synthesis and characterization of the molecular and polymeric structure of all five COFs, their catalytic efficiency in CO_2_RR was investigated. The choice for a mixed Ni/Zn system was based on the possibility of hydride transfer from Ni–H (intermediate during the HER) to Zn–CO sites, where CO could then be reduced further. The results demonstrate that the pure Ni_100_/Zn_0_ and Ni_0_/Zn_100_ COFs favor the formation of H_2_ and CO, respectively. Remarkably, apart from H_2_ and CO reaction products, the Ni_50_/Zn_50_ COF catalyst generates formate (FE = 43%) and CH_4_ (FE = 14%) at 150 mA cm^–2^, showing a distinct catalytic difference from the pure component COFs. Here, the novelty of this research lies not in achieving the highest absolute catalytic efficiencies but rather in the discovery of catalytic synergy between two different metalloporphyrin centers.

## 2 Results

### 2.1 Metalloporphryin COF Structure Investigation

**Figure 1:**
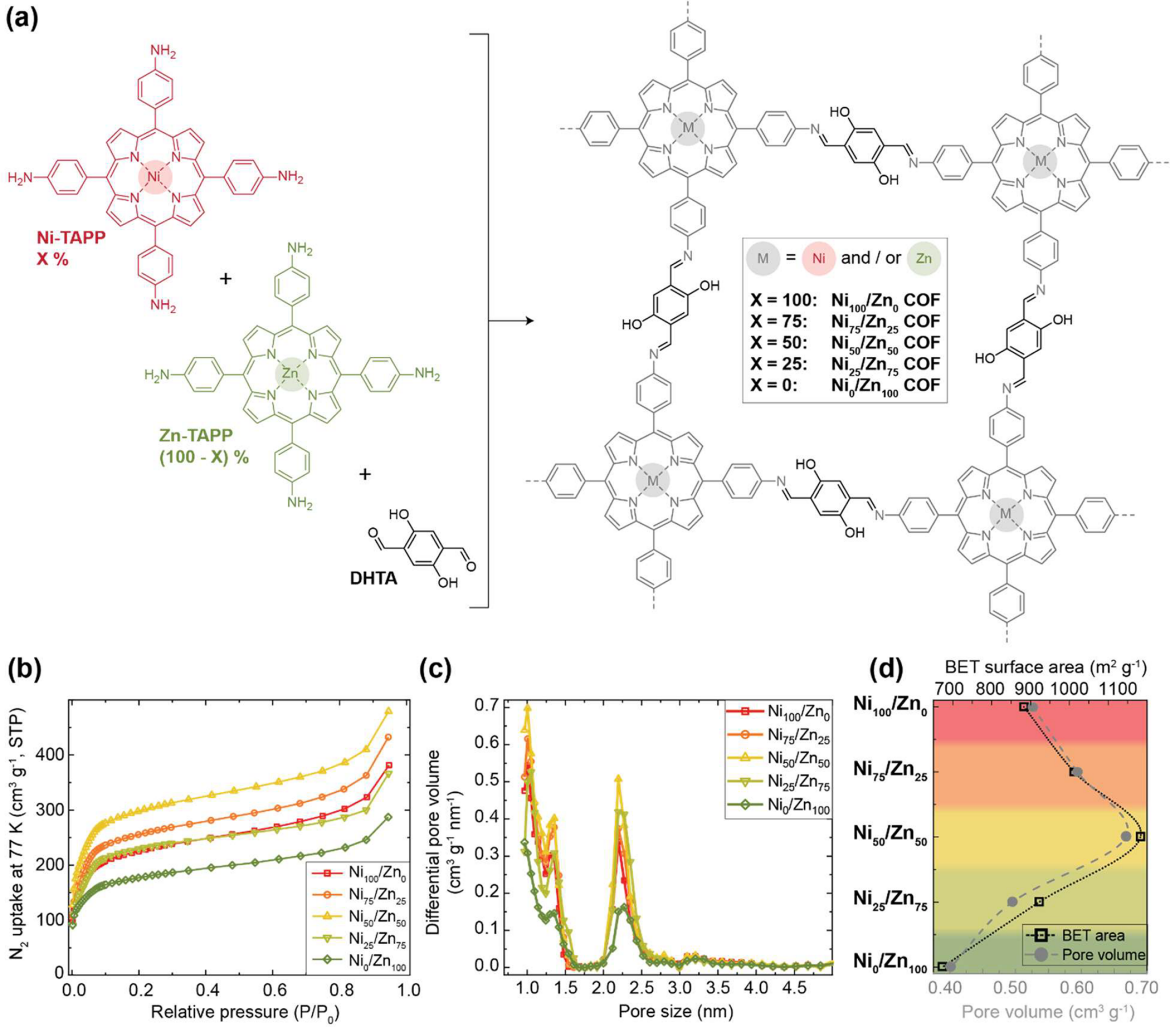
(a) Chemical structure of Ni/Zn-porphyrin COFs and their monomers. (b) Nitrogen adsorption isotherms of Ni/Zn-porphyrin COFs measured at 77 K. (c) Pore size distributions of Ni/Zn-porphyrin COFs, calculated from experimental N_2_ adsorption isotherm branches and based on a QSDFT carbon model with slit/cylindrical pore geometries. (d) BET surface areas and Gurvich total pore volumes of Ni/Zn-porphyrin COFs, calculated from experimental N_2_ adsorption isotherms.

Nickel- and zinc-containing 5,10,15,20-tetra(4-aminophenyl)porphyrin monomers (Ni-TAPP and Zn-TAPP) were synthesized via a 3-step route using commercial building blocks and subsequently analyzed (Scheme S1, Figure S1, and the Experimental Section). The polycondensation reactions of Ni- and/or Zn-TAPP with 2,5-dihydroxyterephthaldehyde (DHTA) under solvothermal conditions yielded COF structures with various ratios of nickel- and zinc porphyrins (Figure 1a). FT-IR spectroscopy, UV–vis spectroscopy, and TGA analysis (Figures S2–S5) were utilized to confirm the formation of these COF structures. The thermal properties of all COFs are similar. The mid-IR and UV–vis spectra of the COFs containing both Ni- and Zn-porphyrins appear to be intermediate between those of the two pure component COFs and follow a clear trend depending on the Ni:Zn ratio.

The nitrogen adsorption isotherms are shown in Figure 1b, and Figure S6. Although Ni/Zn-porphyrin COFs have different porosities depending on the ratio between originating Ni- and Zn-porphyrin monomers, their nitrogen isotherm curve shapes remain similar, following typical curves representative of micro- and small mesoporosity. Interestingly, the Ni_50_/Zn_50_ COF appears to have the largest absolute micro- and mesopore volume. To expand on this, pore size distributions (PSDs) were calculated (Figure 1c). As seen here and Figures S7 and S8, all COFs contain a significant micropore volume (0.17–0.25 cm^3^·g^–1^) and small mesopore volume (0.06–0.13 cm^3^·g^–1^). Finally, surface areas based on BET theory using the BETSI program were calculated (Figures S9–S13),^19^ as well as the Gurvich total pore volume for all COFs, and these values are depicted in the diagram in Figure 1d as a function of the ratio of Ni- and Zn-porphyrin units. An apparent optimum for both properties is seen in the Ni_50_/Zn_50_ COF, having a BET surface area of 1180 m^2^·g^–1^ and a total pore volume of 0.67 cm^3^·g^–1^.

X-ray photoelectron spectroscopy (XPS) was utilized to investigate the surface chemistry of the Ni/Zn-porphyrin COF powders (Figure S14) and atomic percentages of Ni and Zn based on these spectra were calculated (Table S1). The measured Ni:Zn ratios resemble the expected values based on the intended incorporation of Ni- and Zn-porphyrin units during COF synthesis, with slight deviations: 65:35, 40:60, and 29:71 for the Ni_75_/Zn_25_, Ni_50_/Zn_50_, Ni_25_/Zn_75_ COFs, respectively. The polymer network was further investigated by powder X-ray diffraction (PXRD, Figure S15). Short range crystallographic order was detected for all COFs, and the diffractograms indicate a minimal distance of either 2.45 nm (in-plane) or 0.4 nm (interlayer) between neighboring metalloporphyrin sites. We observed correlations between this short-range order and the significant fraction of small mesopores (Figure 1c) identified through N_2_ sorption measurements. On the other hand, the micropore volume likely originates from a combination of interlayer porosity and amorphous segments throughout the polymer network.

**Figure 2:**
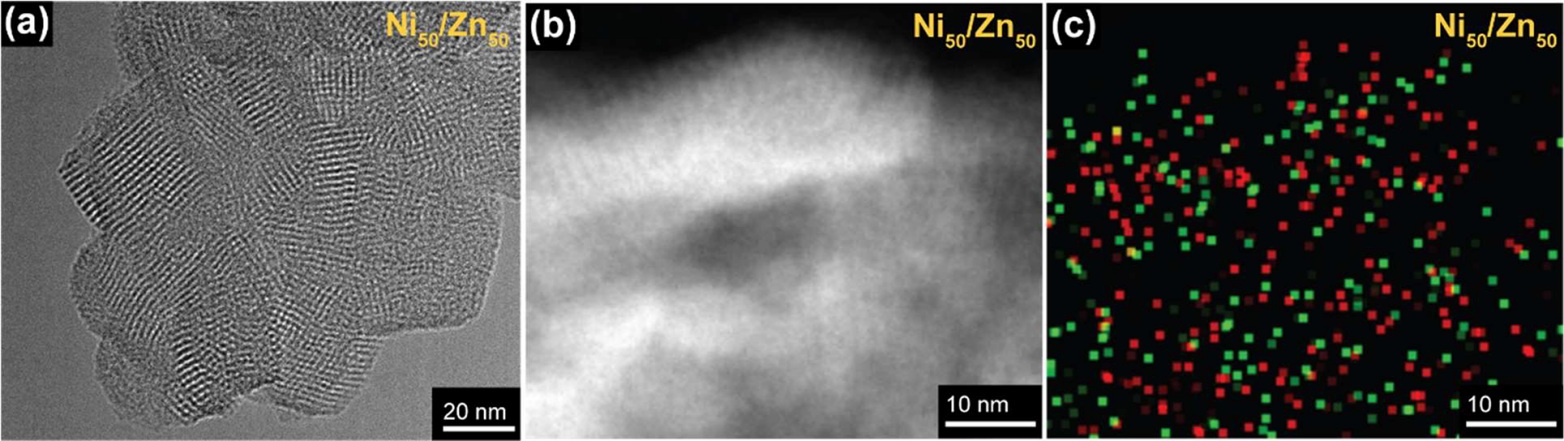
(a) High-resolution TEM image of Ni_50_/Zn_50_-porphyrin COF. (b) STEM-HAADF and (c) STEM-EDX mapping (red representing nickel and green representing zinc) images of Ni_50_/Zn_50_ COF.

The sheet-like character of the COFs was further inspected using high-resolution transmission electron microscopy (HR-TEM). The morphology of all COFs revealed interconnected/aggregated sheets with sizes in the range of 20–40 nm (Figure S16), of which a representative image (Ni_50_/Zn_50_) is shown in Figure 2a. The expected square-geometry of the repeating unit of ∼2 nm is visible in these images. The typical square lattice that was observed for all COF structures, was subsequently related to the proposed molecular structure of the porphyrin-COFs (Figure S17). The distance of repeating units based on PXRD was also indicated here, showing a clear correlation between our PXRD and HR-TEM findings. Closer inspection of the sheet-like structure of the Ni_50_/Zn_50_ COF was performed using scanning transmission electron microscopy (STEM) with a high-angle annular dark field (HAADF) detector, in combination with energy-dispersive X-ray (EDX) spectroscopy (Figure 2b,c). Focusing on the elements Ni and Zn allowed us to obtain a 2D projection of the spatial distribution of these elements throughout the COF structure. Although no actual atomic resolution can be reached (partly due to COF degradation under the beam after a prolonged time), the STEM-EDX map does indicate a relatively homogeneous distribution of Ni- and Zn-porphyrin units with no significant preference to form microdomains of either of the two metallic elements.

### 2.2 Electrochemical CO_2_ Reduction Performance

**Figure 3:**
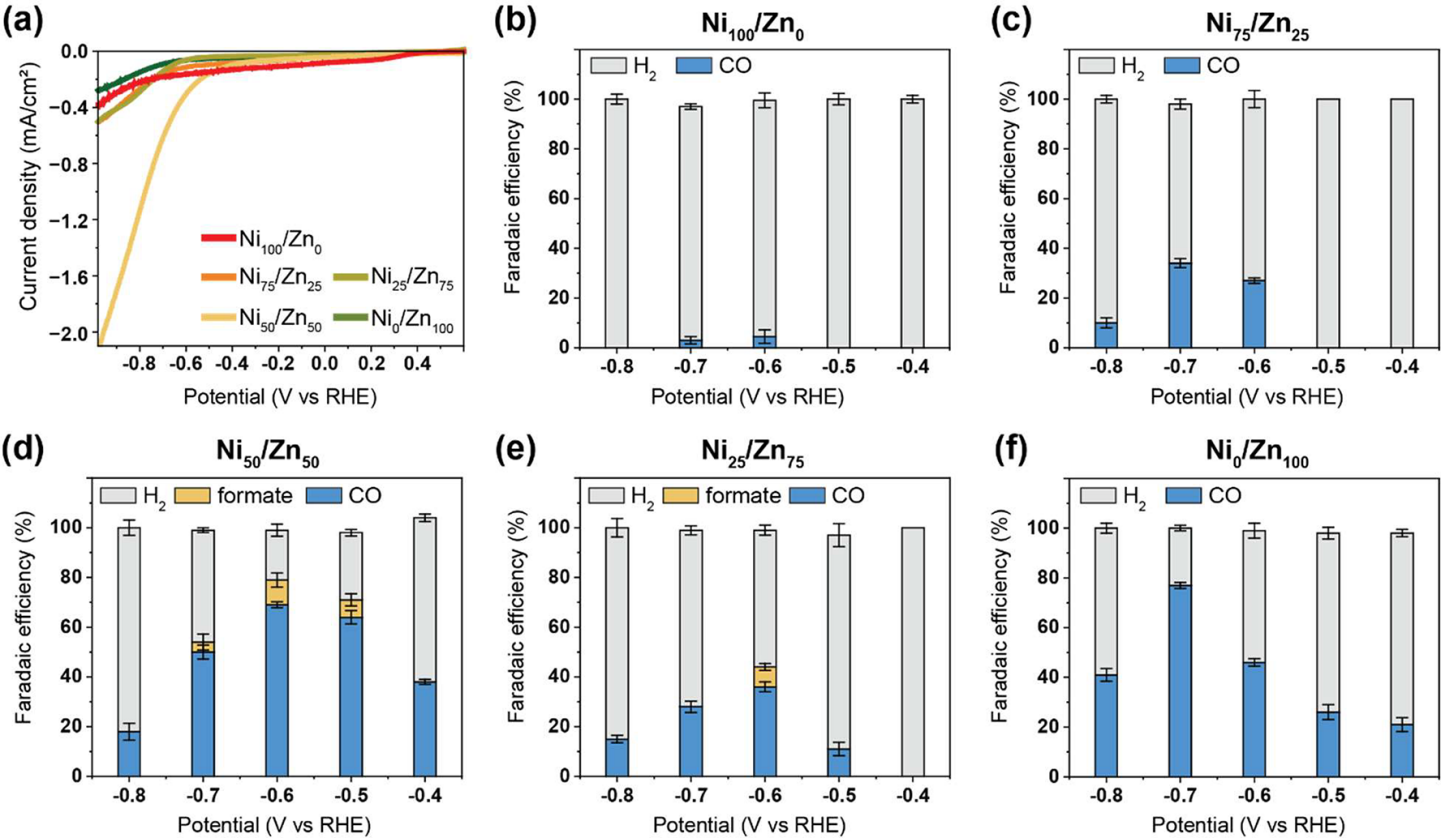
H-cell results for the electrochemical reduction of CO_2_. (a) Linear sweep voltammetry (LSV) comparison of heterogeneous COF complexes. Faradaic efficiency (FE) of (b) Ni_100_/Zn_0_, (c) Ni_75_/Zn_25_, (d) Ni_50_/Zn_50_, (e) Ni_25_/Zn_75_, and _(_f) Ni_0_/Zn_100_ at −0.4 to −0.8 V vs RHE in 0.1 M KHCO_3_.

The electrocatalytic properties of the synthesized COFs were studied using linear sweep voltammetry (LSV, Figure 3a, and Figure S18). A two-compartment electrochemical H-cell setup that included a three-electrode system, including a silver chloride (Ag/AgCl) reference electrode, glassy carbon working electrode (GCE), and a platinum counter electrode, was used for these experiments. To gain insight into the catalytic activity and selectivity of the synthesized catalysts, chronoamperometry was performed at several potentials ranging from −0.4 to −0.8 V vs RHE in CO_2_-saturated 0.1 M KHCO_3_ (Figure S19). In addition, the reduced gaseous and liquid products were periodically sampled from the cathodic chamber headspace and the electrolyte, respectively, and analyzed by gas chromatography (GC), high-performance liquid chromatography (HPLC), and NMR (Figures S20 and S21).

The optimal overpotential (determined from the LSV curve onset) for CO_2_RR on Ni_0_/Zn_100_ and Ni_75_/Zn_25_ was found to be −0.7 V vs RHE, while a more anodic potential of −0.6 V vs RHE was observed in the cases of Ni_100_/Zn_0_, Ni_25_/Zn_75_ and Ni_50_/Zn_50_ (Figure 3, Table S2). The largest increase in current density coupled with a noticeable positive shift to the lowest onset potential (approximately −0.6 V vs RHE) was observed in the case of the Ni_50_/Zn_50_ COF. Aside from H_2_, CO was the sole reduction product from Ni_0_/Zn_100_, Ni_100_/Zn_0_, and Ni_75_/Zn_25_ COFs. In contrast, formate was detected, in addition to CO and H_2_, in the cases of Ni_25_/Zn_75_ and Ni_50_/Zn_50_. Among the five modified electrodes, Ni_50_/Zn_50_ exhibited the highest CO_2_RR FE of 79% (FE_CO_ = 69% and FE_formate_ = 10%), followed by Ni_0_/Zn_100_ (FE_CO_ = 46%), Ni_25_/Zn_75_ (FE_CO_ = 36% and FE_formate_ = 8%), Ni_75_/Zn_25_ (FE_CO_ = 27%), and Ni_100_/Zn_0_ (FE_CO_ = 4.5%) at −0.6 V vs RHE. Turnover number (TON) and turnover frequency (TOF) values are calculated for the CO_2_-converting catalysts (Table S3), yielding a TOF of ∼3000 h^–1^ for the best performing catalyst Ni_50_/Zn_50_, highlighting that these COFs have a high catalytic activity.

Next, for a better understanding of the reaction kinetics of the synthesized catalysts, Tafel plots were generated within the overvoltage window ranging from 0.4 to 0.8 V (Figure S22). The measured values were 212, 184, 92, and 385 mV/dec for Ni_75_/Zn_25_, Ni_50_/Zn_50_, Ni_25_/Zn_70_, and Ni_0_/Zn_100_, respectively. The smallest Tafel slope was observed in the case of bimetallic COFs when the two metal centers are combined, which is possibly linked to facilitated electron transfer through their synergistic effect, in contrast to the monometallic Ni_0_/Zn_100_.

**Figure 4:**
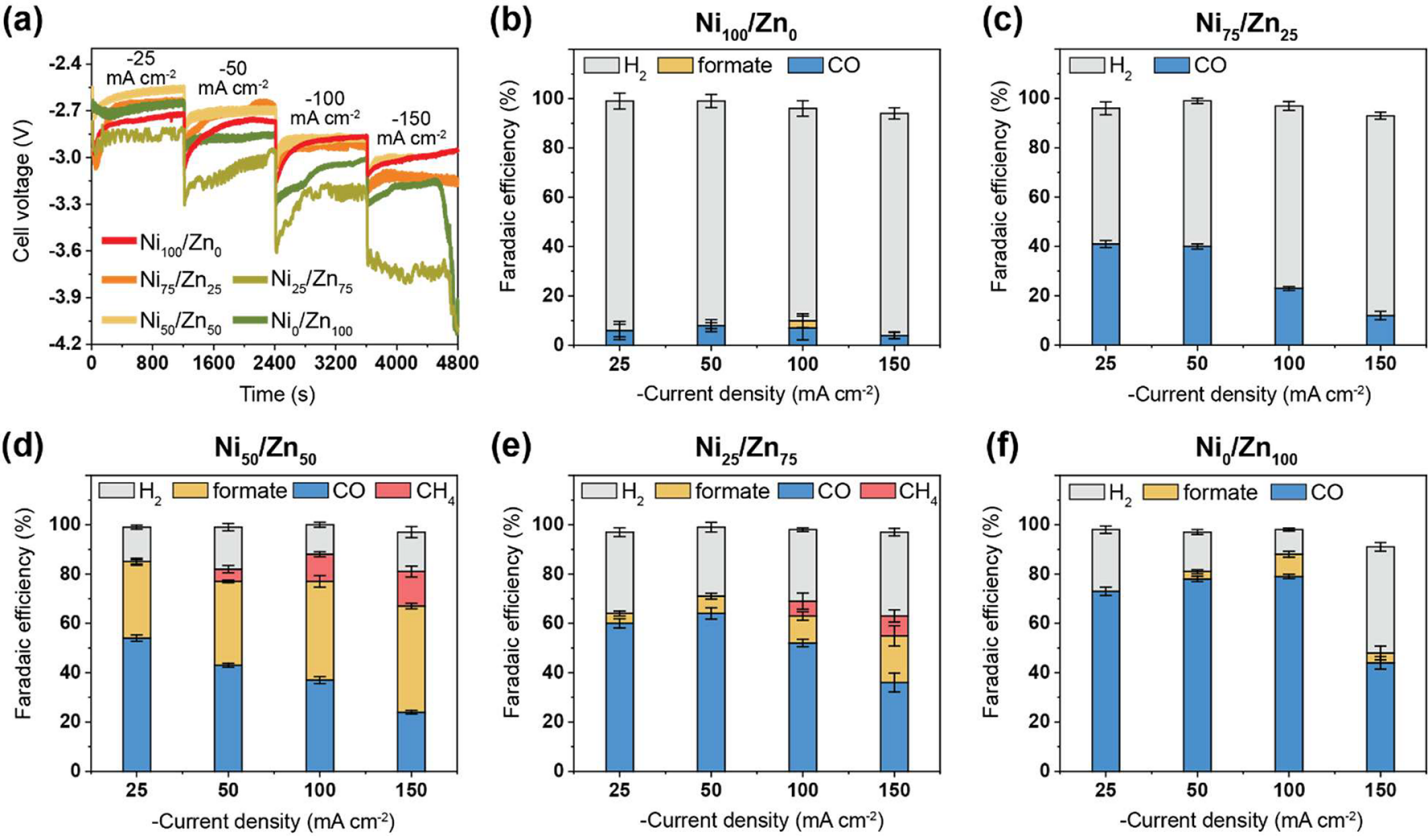
MEA-cell results for the electrochemical reduction of CO_2_. (a) Graph of voltage against time at different current steps in the range of −25 to −150 mA cm^–2^ for all investigated COF catalysts. Faradaic efficiency (FE) comparison of (b) Ni_100_/Zn_0_, (c) Ni_75_/Zn_25_, (d) Ni_50_/Zn_50_, (e) Ni_25_/Zn_75_, and (f) Ni_0_/Zn_100_ from −25 to −150 mA cm^–2^ in 0.5 M KOH.

Although H-cells are useful for studying the catalysts’ behavior, their performance is limited by low solubility of CO_2_ in an aqueous solution, competing HER, and low current densities.^20,21^ Maximizing the interaction between the electrolyte and the gas pathway via the catalytic scaffold allows flow cells to overcome mass transport limitations and suppresses the HER.^22^ Therefore, the catalytic activity of the catalysts was investigated using a MEA cell through stepwise constant current densities ranging from 25 to 150 mA cm^–2^ (Figure 4a and Figure S23). A higher content of nickel (Ni_100_ to Ni_50_) seems to be beneficial when high current densities (100 and 150 mA cm^–2^) are applied, since these catalysts show both a stable signal, as well as relatively low cell voltages. In contrast, the cell voltages measured for Ni_25_/Zn_75_ and Ni_0_/Zn_100_ COFs are rather unstable at these higher current densities, which is possibly an effect of the smaller surface areas and pore volume (reduced CO_2_ and product mass transfer) or catalyst conductivity.

The selectivity obtained for the pure component catalysts in the MEA cells (especially at 150 mA cm^–2^) reflect comparable results to the ones obtained in the H-cell setup: Ni_100_/Zn_0_ generating predominantly H_2_ and Ni_0_/Zn_100_ a combination of CO and H_2_ (Figure 4b and 4f). Generally, the COF catalysts in the MEA setup produce more CO than in the H-cell. Of note should be that in both setups, the catalyst matrices lack the presence of conductive agents (e.g., carbon nanotubes), which would increase the electrocatalytic performance even further as we have seen in previous works.^23^ Both pure component catalysts produce trace amounts of formate in MEA cells, of which the production is highest at 100 mA cm^–2^. In contrast, methane is observed as a unique product for the Ni_50_/Zn_50_ and Ni_25_/Zn_75_ catalysts (Figure 4d,e), where a deviation from the linear average of the pure component catalysts is evident in the relatively large production of formate and CH_4_, especially in the case of Ni_50_/Zn_50_.

The selectivity of this catalyst is FE_formate_ = 40% and FE_CH4_ = 11% at 100 mA cm^–2^, and FE_formate_ = 43% and FE_CH4_ = 14% at 150 mA cm^–2^ (Table S3). Larger amounts of formate and CH_4_ are obtained at higher current densities, with a simultaneous decrease in CO production. The observed production of CH_4_ in the MEA cell particularly is an effect of (next to catalyst activity) the cell design, improved mass transport, higher current density, and the electrolyte. Experimentally testing a wider range of electrolytes would further elucidate electrolyte effects on product formation.^24^ Finally, Ni_50_/Zn_50_ and Ni_0_/Zn_100_ catalysts were most effective in suppressing HER, both having a FE_total_ of 88% for CO_2_RR products at 100 mA cm^–2^.

The partial current densities for the reduced products differ significantly for the various Ni–Zn compositions (Figure S24). Considering the best-performing catalyst (Ni_50_/Zn_50_), the partial current density for CH_4_ and formate increased at higher current density. Generally, the CO partial current density peaks decreased when cell voltages are lower than −3.0 V. In contrast, the partial current density for H_2_ production (*j*_H2_) grows monotonically when the cell potential is more negative. Thus, at more negative cell voltages, the HER becomes dominant.

Scanning electron microscopy (SEM) was utilized to study morphological changes to the polymer particles at the surface (Figure S25). The COF particles are clearly visible on the GDE surface, both before and after catalysis. The effect of the electroreduction on these particles is noticeable in the form of unidentified matter partly covering and interpenetrating the COF particles. XPS was also used to assess chemical changes within the COF after catalytic reactions (Figure S26). A clear difference in the low binding energy region was observed, where peaks at 294.4–294.7 eV were only visible after catalysis. These peaks are characteristic for potassium K 2p, which belongs to salt precipitation.^25^ On the other hand, the spectrum for nitrogen, deconvoluted into peaks at 397.5–397.8 eV and 398.7–399.3 eV, is similar for the electrodes before and after catalysis, suggesting (at least partly) retention of stability of both the porphyrin-ring, as well as imine polymer backbone. Also, the signals for Ni 2p and Zn 2p are present before and after catalysis and show no clear changes. Lastly, FT-IR spectra of the electrode surfaces showed a noticeable difference with peaks at 1618 and 1390 cm^–1^, indicating characteristic vibrations of formate species, only visible after catalysis (Figure S27). Thus, while the COF particles remain stable throughout the electrocatalytic experiments presented here, optimization of their surface chemistry would be needed to ensure long-term stability and functionality. Alternatively, chemically and thermally more robust polymer backbones such as polyimides have been proven to withstand similar electrochemical environments for a large amount of cycles.^26^ A potential approach regarding catalyst stability would be to condense the amino-functionalized porphyrin monomers used in the current research with dianhydrides to yield similar polyimides.

**Figure 5:**
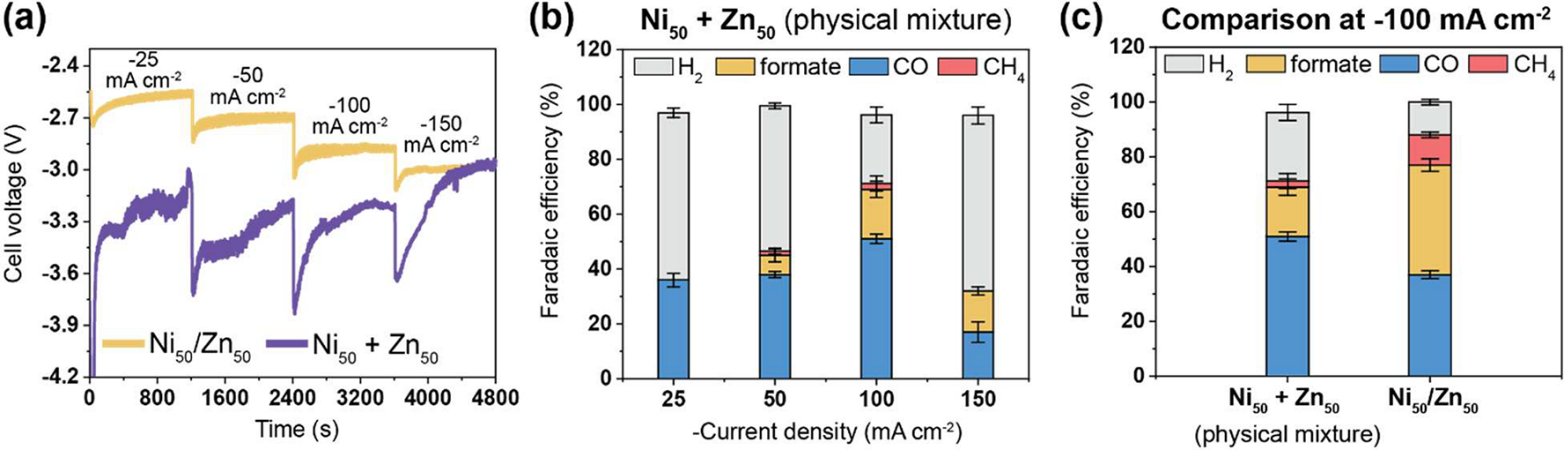
MEA-cell results comparison of Ni_50_/Zn_50_ COF and a 1:1 physical mixture of the Ni_100_/Zn_0_- and Ni_0_/Zn_100_-COFs (named Ni_50_ + Zn_50_) for the electrochemical reduction of CO_2_. (a) Graph of voltage against time at different current steps in the range of −25 to −150 mA/cm^2^. Faradaic efficiency (FE) of (b) Ni_50_ + Zn_50_ and (c) comparison of Ni_50_ + Zn_50_ and Ni_50_/Zn_50_ at −100 mA cm^–2^ in 0.5 M KOH.

To further shed light on the possible catalytic mechanisms using synthesized Ni/Zn COF catalysts, a 1:1 (w/w) physical mixture of Ni_100_/Zn_0_- and Ni_0_/Zn_100_-COFs (named Ni_50_ + Zn_50_) was fabricated by mixing 3.5 mg of each of these COFs in 4 mL of DMF through 40 min sonication. A gas diffusion electrode (GDE) based on this mixture was prepared via the same method as the other COF catalysts (see Experimental Section) and was tested as a control experiment using the same experimental MEA setup. As shown in Figure 5a, Ni_50_ + Zn_50_ exhibited more negative voltages than Ni_50_/Zn_50_. Ni_50_ + Zn_50_ produces higher quantities of H_2_ and more comparable quantities of CO at all current densities than Ni_50_/Zn_50_ (Figure 5b,c). Interestingly, Ni_50_ + Zn_50_ was also able to produce formate and even CH_4_ (FE_formate_ = 18% and FE_CH4_ = 2.2% at 100 mA cm^–2^), albeit at much lower Faradaic efficiencies than Ni_50_/Zn_50_. Apart from the slightly more pronounced formate production and the trace methane quantities, the catalytic activity of Ni_50_ + Zn_50_ resembles the linear average of the Ni_100_/Zn_0_ and Ni_0_/Zn_100_ catalysts quite well.

## 3 Discussion

### 3.1 Structural Synergy in Mixed Nickel- and Zinc-Porphyrin-Based COFs

Utilizing multiple different metalloporphyrin monomers with the same polymerizable functional groups in a single COF synthesis has rarely been explored^14^ and not yet in a systematic manner as has been shown in this research. The chemical differences (Figures S2–S5 and S9) between the five studied frameworks were largely attributed to the implemented ratio of Ni- to Zn-porphyrin units. However, the structural differences in terms of porosity were remarkable, with the Ni_50_/Zn_50_ COF having the highest porosity overall at an apparent optimum regarding the Ni-TAPP: Zn-TAPP ratio (Figure 1d). Regardless of the exact cause for this porosity difference (e.g., monomer geometry and reactivity, interplane and out-of-plane defects, and sheet stacking), here we discovered a yet unexplored design parameter to optimize the COF structure.

Additionally, STEM/EDX mapping did not indicate the presence of microdomains of the two elements but rather a relatively homogeneous spatial distribution of Ni- and Zn-porphyrin units in the mixed metalloporphyrin COFs (Figure 2). As such, the two different active sites are in close (nanometer) proximity with each other, which proves to be important in discussing the plausible mechanisms of reaction product formation during the CO_2_RR experiments.

### 3.2 Catalytic Synergy in Nickel- and Zinc-Porphyrin-Based COFs

As mentioned in the Introduction, while cobalt or zinc metal centers would primarily yield CO as the major product,^14,17^ introducing nickel (with its unique catalytic activity) could enable enhanced proton and electron transfer. This is evidenced by the Ni_50_/Zn_50_ COF catalyst, which successfully produced CO, formate, and CH_4_ during CO_2_RR in the MEA cell. Knowing that single-site nickel and zinc catalysts are generally known for producing H_2_ and CO, respectively,^27−29^ which is also aligned with the selectivity of the pure component catalysts in this work, detecting CH_4_ with transfer of eight electrons and protons is an interesting achievement. We propose three potential pathways of CH_4_ production, beginning with CO as the initial intermediate in the CO_2_reduction reaction (Figure S28). These pathways assume CO production occurs at zinc sites, since the pure component Ni_0_/Zn_100_ catalyst mainly produces CO, whereas the Ni_100_/Zn_0_ catalyst predominantly forms H_2_. Additionally, all pathways consider the retention of framework structural stability under the experimental CO_2_RR conditions, as has been indicated by XPS analysis (Figure S26). Stepwise desorption of CO from zinc, followed by adsorption onto nickel, where it could be converted into CH_4_ is a plausible pathway and similar to the results of other works on stepwise CO_2_ conversion.^18,30^ On the other hand, a likely mechanism could be hydride transfer from Ni-sites (Ni–H complexes being an intermediate in the water splitting reaction) to neighboring Zn–CO sites. Of note should also be the detection of CH_4_ when using a physical mixture of Ni_100_/Zn_0_- and Ni_0_/Zn_100_-COFs, indicating that the observed synergistic effect is likely due to interlayer interaction rather than within a single plane.

To gain additional insights into the mechanism of CO_2_ reduction, DFT calculations were performed to obtain binding energies between CO and Ni(II)- and Zn(II)TPP complexes (Table S5, Figure S29). Ni(II)TPP (high spin state, i.e., its ground state) and Zn(II)TPP show a binding enthalpy of −33.8 kJ/mol, and −29.1 kJ/mol, respectively, suggesting that CO binds more strongly with Ni(II)-TPP than with Zn(II)-TPP. These results add some weight to the earlier proposed mechanism of CO formation on Zn, followed by desorption/adsorption on Ni(II) for further conversion. At the same time, it might still be true that other Ni(II)-centers also participate in the mentioned hydride transfer, possibly not only to Zn(II)-CO complexes, but, taken into account these calculations, also to Ni(II)-CO complexes. However, it is important to note that applying an electrochemical potential, which includes changes in the oxidation state of metalloporphyrins, can significantly affect their binding affinity with CO.^31,32^ Therefore, further DFT studies on charged metalloporphyrins, as well as in situ characterization (e.g., utilizing a spectrochemical H-cell), should be conducted to deepen our understanding.

## 4 Conclusion

A systematic synthetic strategy was developed by combining Ni- and Zn-porphyrin monomers to yield bifunctional COFs (Ni_75_/Zn_25_, Ni_50_/Zn_50_, and Ni_25_/Zn_75_), which were compared to the pure component Ni- and Zn-porphyrin COFs (Ni_100_ and Zn_100_). Structural synergy was discovered as the Ni_50_/Zn_50_ COF exhibited the highest micro- and mesoporosity. Additionally, synergy in CO_2_RR catalytic activity between the two metal centers was apparent through the production of the relative largest amount of CH_4_ using the Ni_50_/Zn_50_ catalyst, compared to mostly H_2_ and CO at the Ni_100_ and Zn_100_ catalysts, respectively. As such, this material platform allowed tunable product selectivity through simple adjustment of the Ni- and Zn-porphyrin monomeric ratio. Considering the wide range of metalloporphyrins available, we anticipate that this strategy will expand the library of bi- or even higher order-functional COF catalysts considerably and that such catalysts are then utilized to tackle complex multistep reactions as is shown here.

## 5 Experimental Section

### Reagents

For synthetic procedures all reagents and solvents were of commercial reagent grade and were used without further purification unless stated otherwise. Pyrrole was purchased from TCI Europe N.V (Zwijndrecht, Belgium). Propionic acid (≥99%), 4-nitrobenzaldehyde (98%), tin(II) chloride dihydrate (98%), acetic anhydride (≥98%), nickel acetate tetrahydrate (98%), and zinc acetate dihydrate (≥98%) were purchased from Merck Sigma (Zwijndrecht, The Netherlands). 1-Butanol and pyridine (≥99%) were purchased from Acros Organics B.V.B.A (Geel, Belgium). Ortho-dichlorobenzene was purchased from abcr GmbH (Karlsruhe, Germany). 2,5-Dihydroxyterephthaldehyde (98%) was purchased from Fluorochem BV (Glossop, United Kingdom). Pyrrole was purified by filtration through a plug of alumina (Acros aluminum oxide, 0.050–0.200 mm, 60A).

### Analysis Techniques

NMR spectra were recorded at 298 K (unless stated otherwise) on an Agilent-400 MR DD2 spectrometer (400 MHz). ^1^H NMR chemical shifts (δ) are given in parts per million (ppm) and were referenced to tetramethylsilane (0.00 ppm). Coupling constants are reported as *J* values in hertz (Hz). Data for ^1^H NMR spectra are reported as follows: chemical shift (multiplicity, coupling constant, integration). Multiplicities are abbreviated as s (singlet) and d (doublet). FT-IR spectra were recorded on a PerkinElmer Spectrum 100 FT-IR spectrometer with a universal ATR accessory over a range of 4000–650 cm^–1^. TGA analyses were performed from 30 to 860 °C under a nitrogen atmosphere at a heating rate of 10 °C·min^–1^ using a PerkinElmer TGA 4000. Prior to the measurement, the samples were degassed at 130 °C for 1 h under a nitrogen atmosphere. Liquid UV–vis spectra were recorded at 298 K on a PerkinElmer Lambda 35 UV–vis spectrometer (quartz cuvette) at a concentration of 5 μM in DMF. Prior to the measurements, the COF-DMF suspensions were sonicated for 30 min at room temperature. Nitrogen isotherms were measured on the NOVAtouch gas sorption analyzer from Quantachrome Instruments with high purity N2 (99.99%) at 77 K. Prior to the sorption measurements, all samples were degassed at 130 °C under vacuum for 16 h. The Quantachrome VersaWin software package was used for calculations of pore size distributions by fitting the nitrogen adsorption isotherms to the quenched solid density functional theory (QSDFT) carbon model (using slit/cylindrical/spherical pores). No smoothing factor was applied for the PSD calculation. X-ray photoemission spectroscopy (XPS) measurements were performed using a monochromatic Al Kα excitation source with a Thermo Scientific K-Alpha spectrometer. The spectrometer was calibrated using the C 1s adventitious carbon with a binding energy of 284.8 eV. The base pressure at the analysis chamber was about 2 × 10^–9^ mbar. The spectra were recorded using a spot size of 400 μm at a pass energy of 50 eV and a step size of 0.1 eV. PXRD patterns were measured on a Rigaku MiniFlex 600 powder diffractometer using a Cu Kα source (λ = 1.5418 Å) over the 2θ range of 2–40° with a scan rate of 1°·min^–1^. For high-resolution transmission electron microscopy analysis, a FEI cubed Cs corrected Titan was used. HREM lattice images are collected on a Thermo Scientific Ceta 16M. A low intensity on the camera was used to avoid beam damage. In scanning mode (STEM) ADF (annular dark field) images are collected. In this mode, a subnanometer beam is scanned on the electron transparent sample and for each beam position the diffracted electrons are collected on a ring shape detector. On heavy/thicker parts of the sample, more diffracted electrons are collected, showing up bright in the image. Elemental mapping in STEM mode was done, using the super-X in the ChemiSTEM configuration. The EDX spectrum is collected for each beam position in a STEM image. The accelerating voltage during STEM and TEM was 300 kV. COF framework degradation was observed after prolonged exposure under this beam. Therefore, images and elemental maps were collected in the first few 1–3 min, before the onset of degradation. For TEM sample preparation, the COF powder was crushed in a mortar first without and then under some ethanol. The dispersion was ultrasonically shaken for 5 min. Using a pipet, the dispersion was drop casted onto a C foil supported with a Cu grid (holey Quantifoil TEM grid). After drying, the grid was ready for TEM inspection. Scanning electron microscopy (SEM) images were recorded with a JEOL JSM-840 SEM: materials were deposited onto a sticky carbon surface on a flat sample holder, vacuum-degassed, and sputtered with gold at a thickness of 15 nm. The data concerning the characterization of the materials described in this work can be accessed and used by others for further studies at 4TU.ResearchData.^33^

### Synthesis of H_2_TNPP (**1a**, Scheme S1)

A solution of 4-nitrobenzaldehyde (18.9 g, 125 mmol, 4.0 equiv) in propionic acid (500 mL) and acetic anhydride (23.6 mL) was heated to 150 °C. Then, pyrrole (8.7 mL, 125 mmol, 4.0 equiv) was added dropwise and the resulting black mixture was refluxed for 30 min at 150 °C. After cooling to rt, the precipitate was successively filtered off, washed with water (200 mL), and dried under high vacuum. Pyridine (200 mL) was added to the black solid, and the suspension was refluxed for 30 min. After cooling to rt, the mixture was stored at 4 °C for 16–20 h. The resulting precipitate was successively filtered off, washed with acetone (200 mL), and dried under high vacuum to afford TNPP (5.5 g, 22%) as a dark purple solid. Due to the poor solubility of H_2_TNPP, it was immediately used in the next step without characterization.

### Synthesis of H_2_TAPP (**1b**, Scheme S1)

H_2_TNPP (5.5 g, 6.9 mmol, 1.0 equiv) was suspended in 37% hydrochloric acid (250 mL), and the resulting mixture was stirred at 20 °C for 30 min. Then, tin(II) chloride dihydrate (23.3 g, 103 mmol, 15 equiv) was added and the reaction mixture was stirred at 80 °C for 30 min. Upon cooling, the mixture was cooled further to 0 °C and carefully neutralized by the addition of ammonium hydroxide (300 mL). The resulting precipitate was filtered off, and air-dried. The black solid was suspended in THF (50 mL) and stirred at 20 °C for 15 min and then it was filtered. Heptane (200 mL) was added to the filtrate to precipitate the product. Most THF was evaporated under reduced pressure and the resulting suspension was centrifuged. The supernatant was removed, the precipitate was washed with pentane (100 mL) and dried under high vacuum to afford H_2_TAPP (1.5 g, 33%) as a purple solid. ^1^H NMR (400 MHz, (CD_3_)_2_SO) δ (ppm): −2.77 (s, 2H), 5.53 (s, 8H), 6.97 (d, *J* = 8.3 Hz, 8H), 7.81 (d, *J* = 8.3 Hz, 8H), 8.84 (s, 8H). Spectral data were in agreement with literature values.^3^

### Synthesis of Ni-TAPP (**2**, Scheme S1)

A solution of H_2_TAPP (530 mg, 0.79 mmol, 1.0 equiv) and nickel(II) acetate tetrahydrate (2.0 g, 7.9 mmol, 10 equiv) in DMF (100 mL) was heated at 100 °C for 16–20 h. After cooling to rt, water (300 mL) was added. The resulting precipitate was filtered off, and air-dried. The residue was dissolved in THF and filtered through a plug of silica. The purified material was dissolved in minimal THF and precipitated by the addition of hexane. Most THF was evaporated under reduced pressure and the resulting suspension was centrifuged. The supernatant was removed, the precipitate was washed with hexane (100 mL) and dried under high vacuum to afford Ni-TAPP (298 mg, 52%) as a red solid. ^1^H NMR (400 MHz, (CD_3_)_2_SO) δ (ppm): 5.46 (s, 8H), 6.88 (d, *J* = 8.4 Hz, 8H), 7.60 (d, *J* = 8.3 Hz, 8H), 8.73 (s, 8H). Spectral data were in agreement with literature values.^3^

### Synthesis of Zn-TAPP (**3**, Scheme S1)

A solution of H_2_TAPP (506 mg, 0.75 mmol, 1.0 equiv) and zinc acetate dihydrate (1.6 g, 7.5 mmol, 10 equiv) in chloroform/methanol (100 mL, 1:1, v/v) was refluxed for 16–20 h. After cooling to rt, triethylamine (2 mL) was added and the mixture was evaporated to dryness. The residue was dissolved in THF and filtered through a plug of silica. The purified material was dissolved in minimal THF and precipitated by the addition of hexane. Most THF was evaporated under reduced pressure and the resulting suspension was centrifuged. The supernatant was removed, the precipitate was washed with hexane (100 mL) and dried under high vacuum to afford ZnTAPP (347 mg, 63%) as a green solid. ^1^H NMR (400 MHz, (CD_3_)_2_SO) δ (ppm): 5.41 (s, 8H), 6.93 (d, *J* = 8.3 Hz, 8H), 7.77 (d, *J* = 8.3 Hz, 8H), 8.80 (s, 8H). Spectral data were in agreement with literature values.^3^

### Synthesis of Ni-/Zn-porphyrin COFs

Ni(II)-5,10,15,20-tetrakis(4-aminophenyl)porphyrin (Ni-TAPP) and Zn(II)-5,10,15,20-tetrakis(4-aminophenyl)porphyrin (Zn-TAPP) compounds were synthesized and analyzed, as detailed in the Supporting Information. *X*% Ni-TAPP and (100 – *X*)% Zn-TAPP, combined, totaling 0.04 mmol, were added to a 20 mL prescorched borosilicate ampule. 6 mL of the solvent mixture (1:1 (v/v) mixture of *ortho*-dichlorobenzene and 1-butanol) was added to the ampule, after which it was sonicated for 1 min. Then, 0.08 mmol of 2,5-dihydroxyterephthaldehyde was separately suspended in 1 mL of acetic acid (6 M in water) and 2 mL of solvent mixture and subsequently dropwise added to the ampule. The mixture in the ampule was briefly homogenized and subjected to three freeze–pump–thaw cycles. Lastly, the ampule was flame-sealed and left in an oven at 120 °C for 3 days. The workup of the COFs included washing with THF (∼6 × 10 mL) until the washing solution was clear of color and the COFs were subsequently washed with acetone (3 × 10 mL). Thereafter, the powders were dried at 60 °C in a vacuum oven for 16 h. The yields of the COFs were: Ni_100_/Zn_0_ (34.0 mg, 86%), Ni_75_/Zn_25_ (37.1 mg, 93%), Ni_50_/Zn_50_ (33.1 mg, 84%), Ni_25_/Zn_75_ (32.7 mg, 82%), Ni_0_/Zn_100_ (33.6 mg, 84%). All COFs appear as light fluffy powders, with colors ranging from Ni_100_/Zn_0_ (deep red), Ni_75_/Zn_25_ (dark red-brown), Ni_50_/Zn_50_ (dark brown), Ni_25_/Zn_75_ (dark brown-green), Ni_0_/Zn_100_ (deep green). A complete overview of the analysis techniques and technique-specific sample preparations are detailed in the Supporting Information.

### Preparation of Deposited COF Complexes onto Electrodes

The mixture of each COF compound (7 mg) in DMF (4 mL) with 5 wt % Nafion was sonicated for 40 min to obtain a well-mixed suspension. Then, the mixture was stirred at room temperature overnight and subsequently drop-casted onto a gas diffusion electrode (GDE, Sigracet 38 BC, 5% PTFE applied nonwoven carbon paper with a microporous layer; 2.5 cm × 2.5 cm) for the membrane electrode assembly (MEA) study. For the H-cell setup, 10 μL of the prepared suspension was drop-casted on the preprepared surface (d = 3.0 mm) of a standard glassy carbon electrode and let to dry for 24 h. All potentials were reported versus the Ag/AgCl reference electrode. Potentials were changed from Ag/AgCl (3 M KCl) to the reversible hydrogen electrode (RHE, *E*_RHE_ = *E*_Ag/AgCl_ + 0.059 × pH + 0.210).

### Characterizations during Electroreduction

The reduced products observed in the cathodic compartment were periodically collected from the reaction headspace and tested by gas chromatography (GC). The concentration of gaseous products (CO, CH_4_, H_2_) was obtained from GC, and the average of 4 injections was used to calculate their Faradaic efficiencies. The gas product from CO_2_ electroreduction was analyzed using a chromatograph (InterScience PerkinElmer Clarus 680) coupled with two thermal conductivity detectors (TCD) and a flame ionization detector (FID), while the liquid product was analyzed using HPLC (Infinity 1260 II LC, Agilent Technologies, Hi-Plex H column (at 50 °C) with VWD (at 210 and 280 nm) and RID (at 40 °C)) (Figures S12 and S13). ^1^H NMR was measured using a Bruker 400 MHz setup and the data were processed in MestreNova. The chemical shifts (δ) are reported in ppm.

### H-Cell and Membrane Electrode Assembly (MEA) Experiments

To evaluate the electroactivity of the synthesized COF complexes, the electrochemical reduction of CO_2_ was first studied with an H-cell using the linear sweep voltammetry (LSV) technique. The two-compartment H-cell comprised a three-electrode configuration, including the immobilized COF catalysts on a glassy carbon working electrode (GCE), a silver/silver chloride (Ag/AgCl) reference electrode, and a platinum (Pt) counter electrode in a CO_2_-saturated 0.1 M KHCO_3_ aqueous solution. Gas-phase products were collected from the reaction headspace and measured using gas chromatography (GC). For experiments with higher current densities, a membrane electrode assembly (MEA) electrolyzer consisting of an anode chamber (Ni-foam anode, Recemat BV) with a liquid phase anolyte (0.5 M KOH) and a cathode chamber (COF on GDE) with a gas phase inlet was employed (schematic shown at Figure S23). The membrane that separates these chambers is a Sustainion anion-exchange membrane (X37-50 grade RT). In this design, gaseous CO_2_ is delivered directly (at 40 mL min^–1^, STP) to the active materials through an inlet located at the back side of the GDE.

### Faradaic Efficiency Calculation (for Both H-Cell and MEA)

Gas phase mole fractions were determined using GC injections periodically (and averaged over 4 times) every 5 min during electrolysis (after stabilization periods). Liquid mole fractions were determined using NMR analysis. To estimate the Faradaic efficiency of gaseous products, the mole fractions of CO and H_2_ were calculated from GC injections. Under constant pressure and temperature (ideal gas law), the volume fraction of the gas products (from GC) equals their corresponding mole fraction. The amount of water vapor exiting the reactor was measured using a humidity sensor and found to be 78% relative humidity, which corresponds to a mole fraction of water of 2.3% (*x*_H2O_ = 0.023). Since the sum of mole fractions is equal to 1, the mole fraction of CO_2_ exiting was calculated as eq 1.
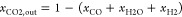
1After calculating the mole fractions of all gaseous products, the volumetric flow rate at the reactor outlet (sccm units) was measured with a mass flow meter and used to calculate the moles of each product.
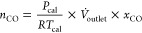
2
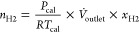
3
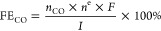
4e.g., *n*_CO_ is mol/s of CO produced, *n*^e^ is the number of electrons involved in CO_2_RR (2 for CO), *F* is 96485 C mol^–1^, and *I* is the applied current (in amperes). *T*_cal_ and *P*_cal_ refer to the calibration *T* and *P* of the mass flow meters.

### Computational Study

DFT calculations were done using version 5.0.4 of the ORCA package^34^ and the PBE0 functional^35^ with Grimme’s D3 dispersion correction with Becke-Johnson damping^36^ using the def2 family of basis sets.^37^ The solvation model based on density (SMD)^38^ was applied to simulate implicit water around the molecules. The calculations were done on the Delft Blue super computer.^39^ First, the structures were optimized at def2-SVP level of theory. Then, single-point calculations and frequency calculations were done at def2-SVP level with the def2-TZVPP basis set on the metal atoms to obtain the energies and check that the structure is at an energetic minimum. Bond enthalpies between CO and MTPP were obtained by comparing the enthalpies of the optimized MTPP-CO, MTPP and CO structures. Calculations for NiTPP and NiTPP-CO complexes were performed in both singlet and triplet states to account for low spin (LS) or high spin (HS) ground states, while ZnTPP and ZnTPP-CO complexes as well as CO were kept in singlet state.

## Supporting Information Available

^1^H NMR and FT-IR of porphyrin monomers; FT-IR, UV–vis, PXRD, TGA, N_2_ sorption including analysis, XPS and HR-TEM of COFs; electrochemical analysis of COF electrodes; SEM, FT-IR and XPS of COF electrode surfaces (PDF)

## References

[ref1] Olah G. A.; Prakash G. K. S.; Goeppert A. Anthropogenic Chemical Carbon Cycle for a Sustainable Future. J. Am. Chem. Soc. 2011, 133, 12881–12898. 10.1021/ja202642y. 21612273

[ref2] Qiao J.; Liu Y.; Hong F.; Zhang J. A review of catalysts for the electroreduction of carbon dioxide to produce low-carbon fuels. Chem. Soc. Rev. 2014, 43, 631–675. 10.1039/C3CS60323G. 24186433

[ref3] Abdinejad M.; Irtem E.; Farzi A.; Sassenburg M.; Subramanian S.; Iglesias van Montfort H.-P.; Ripepi D.; Li M.; Middelkoop J.; Seifitokaldani A.; Burdyny T. CO_2_ Electrolysis via Surface-Engineering Electrografted Pyridines on Silver Catalysts. ACS Catal. 2022, 12, 7862–7876. 10.1021/acscatal.2c01654. 35799769 PMC9251727

[ref4] Cheng T.; Xiao H.; Goddard W. A. III Reaction Mechanisms for the Electrochemical Reduction of CO_2_ to CO and Formate on the Cu(100) Surface at 298 K from Quantum Mechanics Free Energy Calculations with Explicit Water. J. Am. Chem. Soc. 2016, 138, 13802–13805. 10.1021/jacs.6b08534. 27726392

[ref5] Abdinejad M.; Tang K.; Dao C.; Saedy S.; Burdyny T. Immobilization strategies for porphyrin-based molecular catalysts for the electroreduction of CO_2_. J. Mater. Chem. A 2022, 10, 7626–7636. 10.1039/D2TA00876A. PMC8981215 35444810

[ref6] Huang X.; Zhang Y.-B. Reticular materials for electrochemical reduction of CO_2_. Coord. Chem. Rev. 2021, 427, 213564 10.1016/j.ccr.2020.213564.

[ref7] Diercks C. S.; Liu Y.; Cordova K. E.; Yaghi O. M. The role of reticular chemistry in the design of CO_2_ reduction catalysts. Nat. Mater. 2018, 17, 301–307. 10.1038/s41563-018-0033-5. 29483634

[ref8] Zhan T.; Zou Y.; Yang Y.; Ma X.; Zhang Z.; Xiang S. Two-dimensional Metal-organic Frameworks for Electrochemical CO_2_ Reduction Reaction. ChemCatChem 2022, 14, e20210145 10.1002/cctc.202101453.

[ref9] Chen X.; Addicoat M.; Jin E.; Zhai L.; Xu H.; Huang N.; Guo Z.; Liu L.; Irle S.; Jiang D. Locking Covalent Organic Frameworks with Hydrogen Bonds: General and Remarkable Effects on Crystalline Structure, Physical Properties, and Photochemical Activity. J. Am. Chem. Soc. 2015, 137, 3241–3247. 10.1021/ja509602c. 25706112

[ref10] Yuan J.; Chen S.; Zhang Y.; Li R.; Zhang J.; Peng T. Structural Regulation of Coupled Phthalocyanine–Porphyrin Covalent Organic Frameworks to Highly Active and Selective Electrocatalytic CO_2_ Reduction. Adv. Mater. 2022, 34, 2203139 10.1002/adma.202203139. 35654012

[ref11] Abdinejad M.; Seifitokaldani A.; Dao C.; Sargent E. H.; Zhang X.-A.; Kraatz H. B. Enhanced Electrochemical Reduction of CO_2_ Catalyzed by Cobalt and Iron Amino Porphyrin Complexes. ACS Appl. Energy Mater. 2019, 2, 1330–1335. 10.1021/acsaem.8b01900.

[ref12] Gong Y.-N.; Zhong W.; Li Y.; Qiu Y.; Zheng L.; Jiang J.; Jiang H.-L. Regulating Photocatalysis by Spin-State Manipulation of Cobalt in Covalent Organic Frameworks. J. Am. Chem. Soc. 2020, 142, 16723–16731. 10.1021/jacs.0c07206. 32894021

[ref13] Qian Y.; Li D.; Han Y.; Jiang H.-L. Photocatalytic Molecular Oxygen Activation by Regulating Excitonic Effects in Covalent Organic Frameworks. J. Am. Chem. Soc. 2020, 142, 20763–20771. 10.1021/jacs.0c09727. 33226795

[ref14] Lin S.; Diercks C. S.; Zhang Y.-B.; Kornienko N.; Nichols E. M.; Zhao Y.; Paris A. R.; Kim D.; Yang P.; Yaghi O. M.; Chang C. J. Covalent organic frameworks comprising cobalt porphyrins for catalytic CO_2_ reduction in water. Science 2015, 349, 1208–1213. 10.1126/science.aac8343. 26292706

[ref15] Diercks C. S.; Lin S.; Kornienko N.; Kapustin E. A.; Nichols E. M.; Zhu C.; Zhao Y.; Chang C. J.; Yaghi O. M. Reticular Electronic Tuning of Porphyrin Active Sites in Covalent Organic Frameworks for Electrocatalytic Carbon Dioxide Reduction. J. Am. Chem. Soc. 2018, 140, 1116–1122. 10.1021/jacs.7b11940. 29284263

[ref16] Chen R.; Wang Y.; Ma Y.; Mal A.; Gao X.-Y.; Gao L.; Qiao L.; Li X.-B.; Wu L.-Z.; Wang C. Rational design of isostructural 2D porphyrin-based covalent organic frameworks for tunable photocatalytic hydrogen evolution. Nat. Commun. 2021, 12, 1354 10.1038/s41467-021-21527-3. 33649344 PMC7921403

[ref17] Zhong H.; Ghorbani-Asl M.; Hoang Ly K.; Zhang J.; Ge J.; Wang M.; Liao Z.; Makarov D.; Zschech E.; Brunner E.; Weidinger I. M.; Zhang J.; Krasheninnikov A. V.; Kaskel S.; Dong R.; Feng X. Synergistic electroreduction of carbon dioxide to carbon monoxide on bimetallic layered conjugated metal-organic frameworks. Nat. Commun. 2020, 11, 1409 10.1038/s41467-020-15141-y. 32179738 PMC7075876

[ref18] Qiu X.-F.; Zhu H.-L.; Huang J.-R.; Liao P.-Q.; Chen X.-M. Highly Selective CO_2_ Electroreduction to C_2_H_4_ Using a Metal–Organic Framework with Dual Active Sites. J. Am. Chem. Soc. 2021, 143, 7242–7246. 10.1021/jacs.1c01466. 33956435

[ref19] Osterrieth J. W. M.; Rampersad J.; Madden D.; Rampal N.; Skoric L.; Connolly B.; Allendorf M. D.; Stavila V.; Snider J. L.; Ameloot R.; Marreiros J.; Ania C.; Azevedo D.; Vilarrasa-Garcia E.; Santos B. F.; Bu X.; Chang Z.; Bunzen H.; Champness N. R.; Griffin S. L.; Chen B.; Lin R.; Coasne B.; Cohen S.; Moreton J. C.; Colón Y. J.; Chen L.; Clowes R.; Coudert F.; Cui Y.; et al. How Reproducible are Surface Areas Calculated from the BET Equation?. Adv. Mater. 2022, 34, 2201502 10.1002/adma.202201502. 35603497

[ref20] Dinh C. T.; Burdyny T.; Kibria G.; Seifitokaldani A.; Gabardo C. M.; Pelayo García De Arquer F.; Kiani A.; Edwards J. P.; De Luna P.; Bushuyev O. S.; Zou C.; Quintero-Bermudez R.; Pang Y.; Sinton D.; Sargent E. H. CO_2_ electroreduction to ethylene via hydroxide-mediated copper catalysis at an abrupt interface. Science 2018, 360, 783–787. 10.1126/science.aas9100. 29773749

[ref21] Pang Y.; Burdyny T.; Dinh C.-T.; Kibria M. G.; Fan J. Z.; Liu M.; Sargent E. H.; Sinton D. Joint tuning of nanostructured Cu-oxide morphology and local electrolyte programs high-rate CO_2_ reduction to C_2_H_4_. Green Chem. 2017, 19, 4023–4030. 10.1039/C7GC01677H.

[ref22] Subramanian S.; Yang K.; Li M.; Sassenburg M.; Abdinejad M.; Irtem E.; Middelkoop J.; Burdyny T. Geometric Catalyst Utilization in Zero-Gap CO_2_ Electrolyzers. ACS Energy Lett. 2023, 8, 222–229. 10.1021/acsenergylett.2c02194. 36660371 PMC9841604

[ref23] Abdinejad M.; Dao C.; Deng B.; Dinic F.; Voznyy O.; Zhang X.; Kraatz H. B. Electrocatalytic Reduction of CO_2_ to CH_4_ and CO in Aqueous Solution Using Pyridine-Porphyrins Immobilized onto Carbon Nanotubes. ACS Sustain. Chem. Eng. 2020, 8, 9549–9557. 10.1021/acssuschemeng.0c02791.

[ref24] Yang K.; Kas R.; Smith W. A. In Situ Infrared Spectroscopy Reveals Persistent Alkalinity near Electrode Surfaces during CO_2_ Electroreduction. J. Am. Chem. Soc. 2019, 141, 15891–15900. 10.1021/jacs.9b07000. 31523949 PMC6788196

[ref25] Lee S.; Ju H.; Machunda R.; Uhm S.; Lee J. K.; Lee H. J.; Lee J. Sustainable production of formic acid by electrolytic reduction of gaseous carbon dioxide. J. Mater. Chem. A 2015, 3, 3029–3034. 10.1039/C4TA03893B.

[ref26] Fernando N.; Veldhuizen H.; Nagai A.; van der Zwaag S.; Abdelkader A. Layer-by-Layer Electrode Fabrication for Improved Performance of Porous Polyimide-Based Supercapacitors. Materials 2022, 15, 4 10.3390/ma15010004. PMC8745899 35009150

[ref27] Yuan S.; Cui L.; He X.; Zhang W.; Asefa T. Nickel foam-supported Fe,Ni-Polyporphyrin microparticles: Efficient bifunctional catalysts for overall water splitting in alkaline media. Int. J. Hydrog. Energy 2020, 45, 28860–28869. 10.1016/j.ijhydene.2020.08.013.

[ref28] Lashgari A.; Williams C. K.; Glover J. L.; Wu Y.; Chai J.; Jiang J. Enhanced Electrocatalytic Activity of a Zinc Porphyrin for CO_2_ Reduction: Cooperative Effects of Triazole Units in the Second Coordination Sphere. Chem.—Eur. J. 2020, 26, 16774–16781. 10.1002/chem.202002813. 32701198

[ref29] Abdinejad M.; Wilm L. F. B.; Dielmann F.; Kraatz H. B. Electroreduction of CO_2_ Catalyzed by Nickel Imidazolin-2-ylidenamino-Porphyrins in Both Heterogeneous and Homogeneous Molecular Systems. ACS Sustain. Chem. Eng. 2021, 9, 521–530. 10.1021/acssuschemeng.0c07964.

[ref30] Hu C.; Jiang Z.; Wu Q.; Cao S.; Li Q.; Chen C.; Yuan L.; Wang Y.; Yang W.; Yang J.; Peng J.; Shi W.; Zhai M.; Mostafavi M.; Ma J. Selective CO_2_ reduction to CH_3_OH over atomic dual-metal sites embedded in a metal-organic framework with high-energy radiation. Nat. Commun. 2023, 14, 4767 10.1038/s41467-023-40418-3. 37553370 PMC10409780

[ref31] Swistak C.; Kadish K. M. Electrochemistry of Iron Porphyrins under a Carbon Monoxide Atmosphere. Interactions between Carbon Monoxide and Pyridine. Inorg. Chem. 1987, 26 (3), 405–412. 10.1021/ic00250a014.

[ref32] Mu X. H.; Kadish K. M. Oxidative Electrochemistry of Cobalt Tetraphenylporphyrin under a CO Atmosphere. Interaction between Carbon Monoxide and Electrogenerated [(TPP)Co]+ in Nonbonding Media. Inorg. Chem. 1989, 28 (19), 3743–3747. 10.1021/ic00318a025.

[ref33] Veldhuizen H.; Abdinejad M.; Gilissen P.; Burdyny T.; Tichelaar F. D.; van der Zwaag S.; van der Veen M. A.; Albertsma J. Dataset from “Combining Nickel- and Zinc-Porphyrin Sites via Covalent Organic Frameworks for Electrochemical CO_2_ Reduction”; 4TU.ResearchData, 2023; 10.4121/bac3310c-bfdc-4f0b-a8b3-34849a4eae2d. PMC11231983 38914515

[ref34] Neese F.; Wennmohs F.; Becker U.; Riplinger C. The ORCA Quantum Chemistry Program Package. J. Chem. Phys. 2020, 152 (22), 224108 10.1063/5.0004608. 32534543

[ref35] Adamo C.; Barone V. Toward Reliable Density Functional Methods without Adjustable Parameters: The PBE0 Model. J. Chem. Phys. 1999, 110 (13), 6158–6170. 10.1063/1.478522.

[ref36] Grimme S.; Ehrlich S.; Goerigk L. Effect of the Damping Function in Dispersion Corrected Density Functional Theory. J. Comput. Chem. 2011, 32 (7), 1456–1465. 10.1002/jcc.21759. 21370243

[ref37] Weigend F.; Ahlrichs R. Balanced Basis Sets of Split Valence, Triple Zeta Valence and Quadruple Zeta Valence Quality for H to Rn: Design and Assessment of Accuracy. Phys. Chem. Chem. Phys. 2005, 7 (18), 3297–3305. 10.1039/b508541a. 16240044

[ref38] Marenich A. V.; Cramer C. J.; Truhlar D. G. Universal Solvation Model Based on Solute Electron Density and on a Continuum Model of the Solvent Defined by the Bulk Dielectric Constant and Atomic Surface Tensions. J. Phys. Chem. B 2009, 113 (18), 6378–6396. 10.1021/jp810292n. 19366259

[ref39] Delft High Performance Computing Centre (DHPC). Delft Blue Supercomputer (Phase 2), 2024. https://www.tudelft.nl/dhpc/ark/delftbluephase2.

