# Supplementary material for: Combining Nickel- and Zinc-Porphyrin Sites via Covalent Organic Frameworks for Electrochemical CO2 Reduction
Source: ACS Appl Mater Interfaces. 2024 Jun 24;16(26):34010–9. doi: 10.1021/acsami.4c02511 (PMC11231983; doi:10.1021/acsami.4c02511)
Supplement: Supplementary file 1 — am4c02511_si_001.pdf [file am4c02511_si_001.pdf]

Supporting Information

Combining Nickel- and Zinc-Porphyrin Sites via Covalent Organic Frameworks for Electrochemical CO_2 Reduction

Hugo Veldhuizen†,[a,b] Maryam Abdinejad†,[c] Pieter J. Gilissen,[d] Jelco Albertsma,[b] Thomas Burdyny,[c] Frans D. Tichelaar,[e] Sybrand van der Zwaag,[a] Monique A. van der Veen*[b]*

(a) Novel Aerospace Materials, Faculty of Aerospace Engineering, Technische Universiteit Delft, 2629 HS, Delft, The Netherlands

(b) Catalysis Engineering, Faculty of Applied Sciences, Technische Universiteit Delft, 2629 HZ, Delft, The Netherlands

(c) Materials for Energy Conversion and Storage, Faculty of Applied Sciences, Technische Universiteit Delft, 2629 HZ, Delft, The Netherlands

(d) Molecular Nanotechnology, Institute for Molecules and Materials, Radboud Universiteit, 6525 AJ, Nijmegen, The Netherlands

(e) Kavli Institute of Nanoscience, Quantum Nanoscience, Physics building, Technische Universiteit Delft, 2628 CJ, Delft, The Netherlands

Corresponding Authors

* Monique A. van der Veen: M.A.vanderVeen@tudelft.nl * Thomas Burdyny: T.E.Burdyny@tudelft.nl

Keywords: covalent organic frameworks, Ni- and Zn-porphyrins, CO_2 electroreduction, CO_2RR, electrolysis, bifunctional catalysis

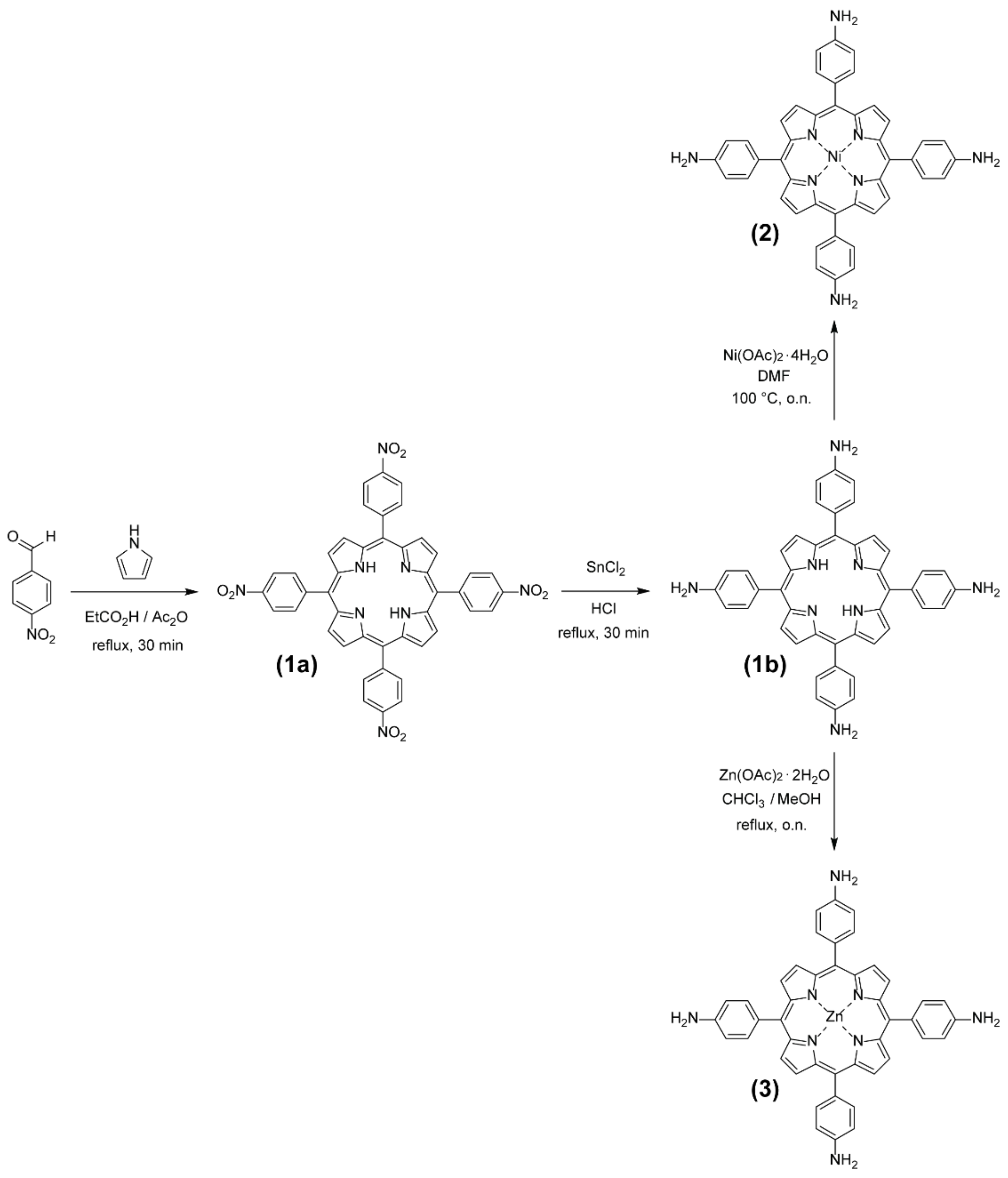

Scheme S1. Reaction scheme towards 5,10,15,20-tetrakis(4-nitrophenyl) porphyrin (**1a**); 2H-5,10,15,20-tetrakis(4-aminophenyl) porphyrin (**1b**); Ni(II)-5,10,15,20-tetrakis(4-aminophenyl) porphyrin (**2**); and Zn(II)-5,10,15,20-tetrakis(4-aminophenyl) porphyrin (**3**).

Proton Nucleic Magnetic Resonance (^1H NMR) and Fourier-transform infrared spectroscopy (FT-IR) spectroscopy confirmed the identity of the products. Comparisons between the ^1H NMR spectra in Figure S1 provides evidence of successful product synthesis. The characteristic singlet from the two pyrrole N-H protons in the porphyrin inner core can be found at −2.7 ppm (Figure S1a), which disappeared after the metal insertion (Figure S1b-S1c), indicating the successful metalation of the porphyrins.

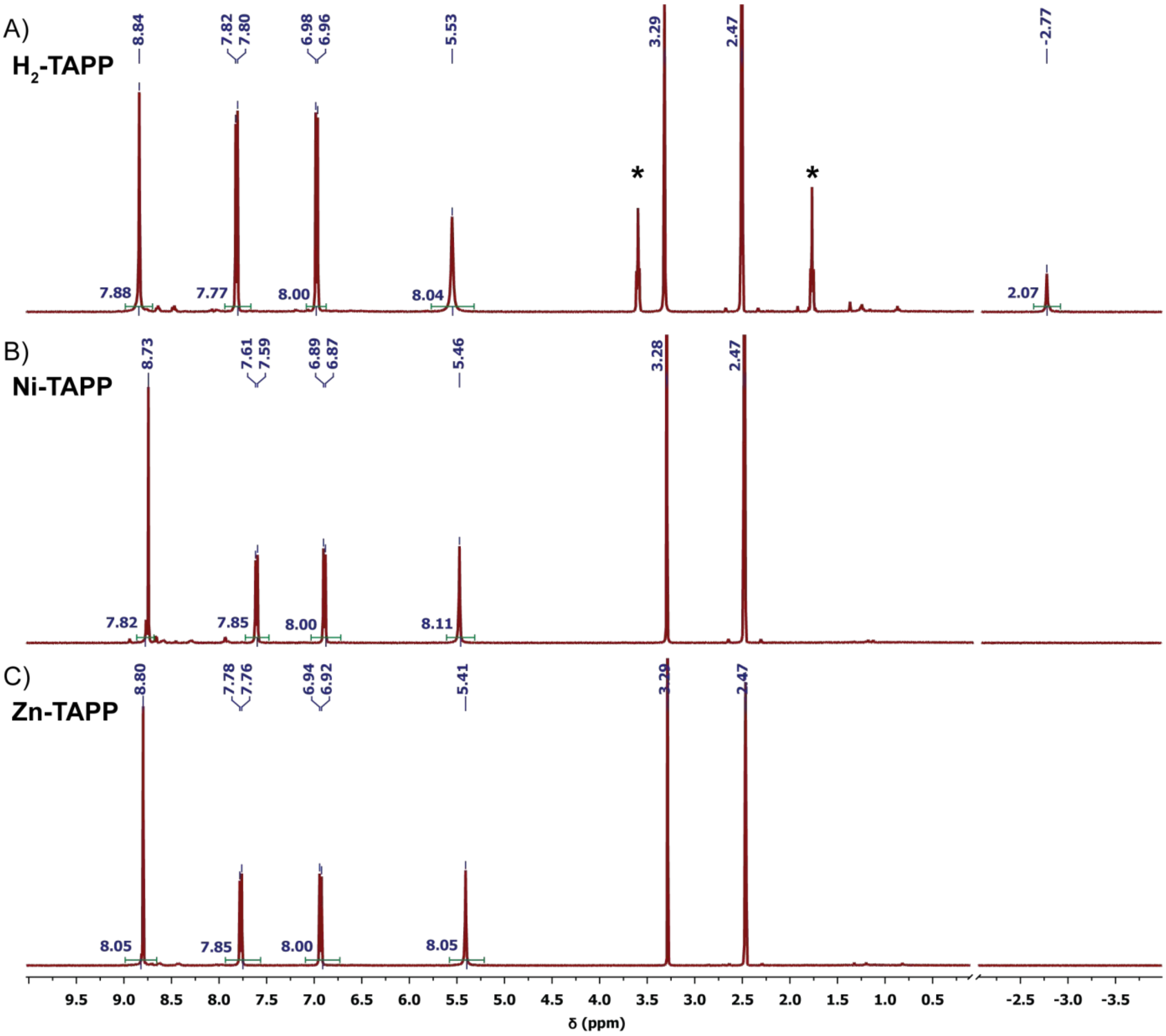

Figure S1. ^1H-NMR spectra of the porphyrin monomers: **A)** H_2TAPP (**1b**), **B)** Ni-TAPP (**2**), and **C)** Zn-TAPP (**3**). The asterisks indicate the presence of residual solvent (THF).

FT-IR measurements further confirmed the formation of the imine polymer backbone (C=N bonds at 1615 cm^{-1}) and the complete disappearance of the monomer functional groups (Figure S2).

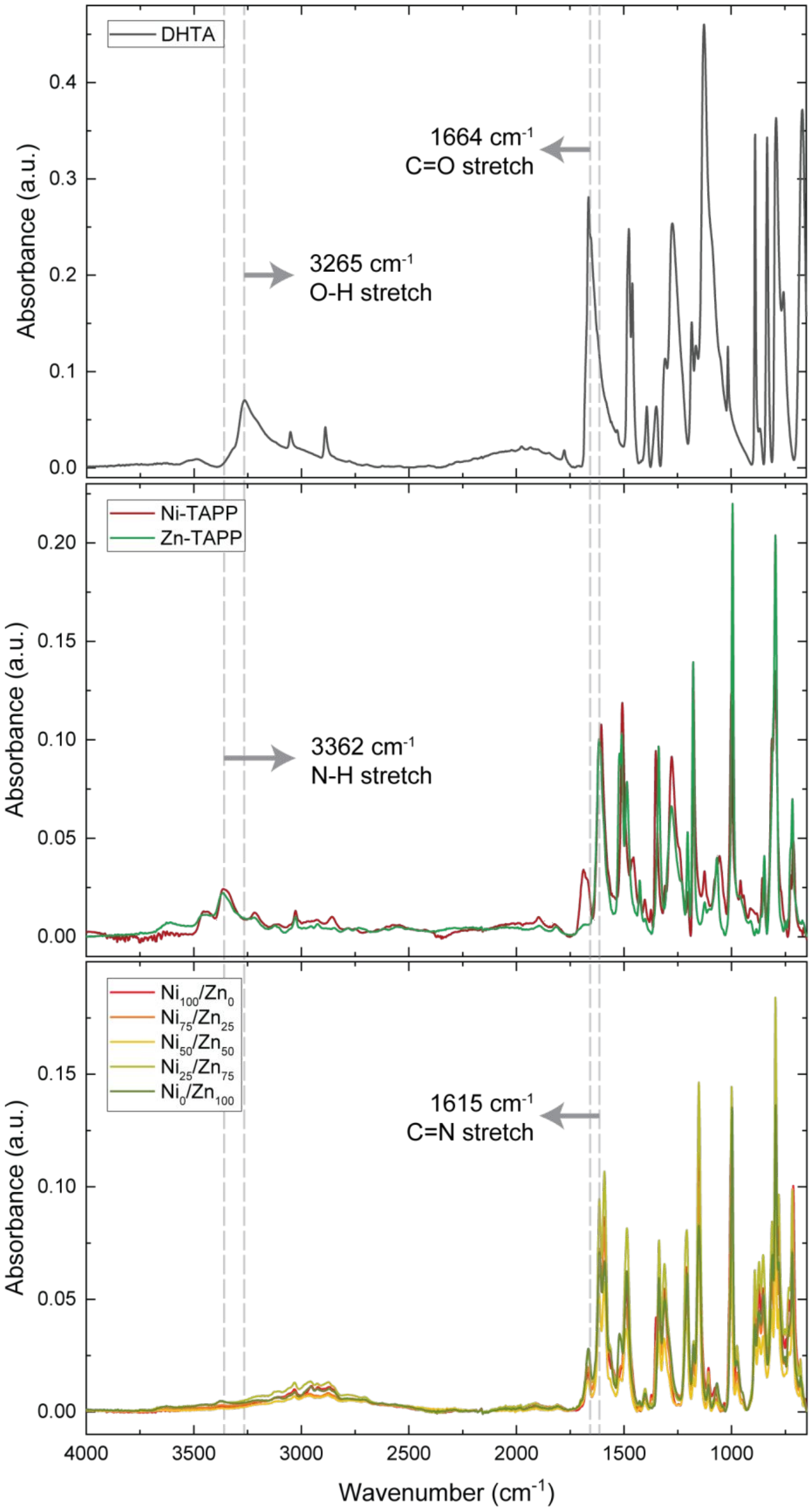

Figure S2. FT-IR spectra of 2,5-dihydroxyterephthaldehyde (DHTA, top), nickel- and zinc-inserted 5,10,15,20-tetrakis(4-aminophenyl)porphyrin (Ni-TAPP and Zn-TAPP, middle) and Ni/Zn-porphyrin COFs (bottom). Characteristic vibrations for each compound are indicated.

The nickel-to-zinc porphyrin ratio influenced the spectroscopic properties, mainly in the fingerprint region of the FT-IR spectra (Figure S3). The rocking vibrational mode of the C-H groups within the pyrrole ring is significantly red-shifted from the Ni_{100}/Zn_0 COF (at 1003 cm-

[1]) to the Ni_0/Zn_{100} COF (at 997 cm^{-1}), as a result of the metal-ligand interaction. In addition, out-of-plane phenyl-ring blue shifts occur from 746 to 751 cm^{-1} and from 713 to 718 cm^{-1} for Ni_{100}/Zn_0 COF to the Ni_0/Zn_{100} COF. The synthesized frameworks that contain both Ni- and Zn-porphyrin units show gradual shifts expected based on their Ni:Zn ratios.

Ni(II)- and Zn(II)-porphyrin units are chemically and geometrically different metal-ligand complexes. The former complex, resembles a square-planar geometry while the Zn(II)-porphyrin complex does not, since the Zn-atom is coordinated to the porphyrin nitrogens at a position slightly above the porphyrin ring.[Ref S1] The metal ion-dependencies of the C-H pyrrole rocking vibration and the out-of-plane phenyl-ring vibrations as described for various metalloporphyrins[Ref S1-Ref S3] are preserved in the porphyrin-based COF structures, and the mixed metalloporphyrin COFs display both Ni- and Zn-characteristic signals.

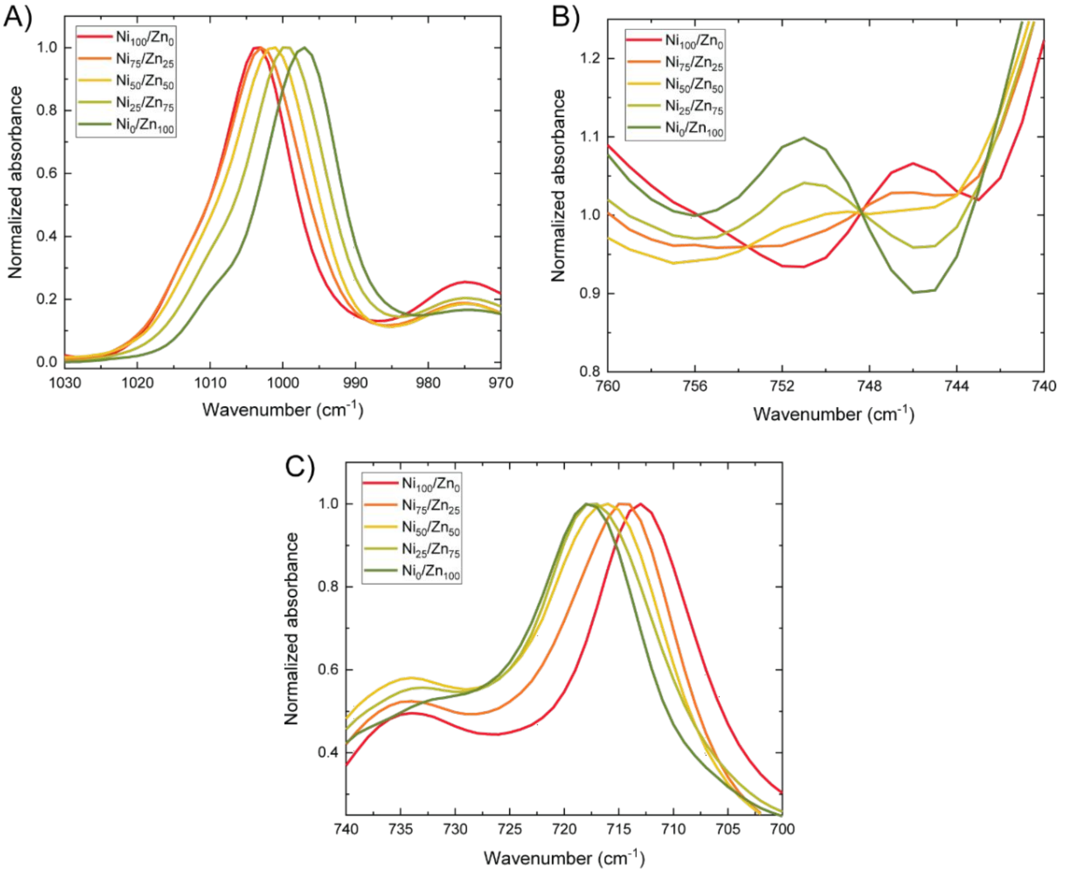

Figure S3. Normalized FT-IR spectra of Ni/Zn-porphyrin COFs, zoomed-in on the region of: **A)** pyrrole rocking vibrations and **B)** and **C)** out-of-plane phenyl- and porphyrin ring-vibrations.

Suspended COF particles were analysed with UV-vis spectroscopy, where metalloporphyrin-characteristic Q-bands were observed (Figure S4). The band at 545 nm is visible for the Ni_{100}/Zn_0 COF but absent for the Ni_0/Zn_{100} COF, while the opposite is true for the band at 624 nm. However, both Q-bands are visible for the co-synthesized metalloporphyrin COFs. In

addition, the characteristic Soret band is located at 433 nm for the Ni_{100}/Zn_0 COF, while it is centred around 437 nm for all other COFs.

The outlier in the UV spectra is the Soret band of the Ni_{100}/Zn_0 COF (at 433 nm instead of 437 nm). The red-shift of the Soret band is attributed to complexation of a porphyrin-coordinated metal with an organic ligand (in this case DMF solvent molecules) at the axial position. This phenomenon is known to occur for Zn(II)-porphyrin units but not for Ni(II),[Ref S4] since the d^8 square-planar geometry of the Ni(II) ion coordinated to the porphyrin ligand is a highly stable conformation. The fact that solvent molecules are able to interact with the COF's active sites (at least with the zinc-ion), highlights the accessibility of these sites, which is promising for their catalytic performance.

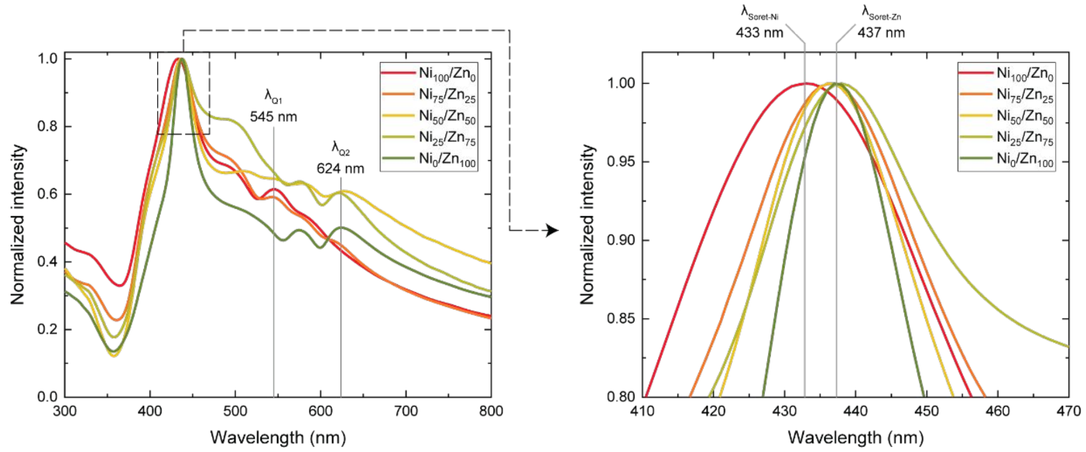

Figure S4. UV-vis spectra of Ni/Zn-porphyrin COF particles sonicated in DMF. The right graph is a zoom-in of the left. Ni- and Zn-porphyrin-characteristic Soret bands and Q bands are indicated.

The presence of the thermally stable imine backbone was also confirmed with TGA analysis (Figure S5), where no significant differences in thermal stability were observed between the COFs.

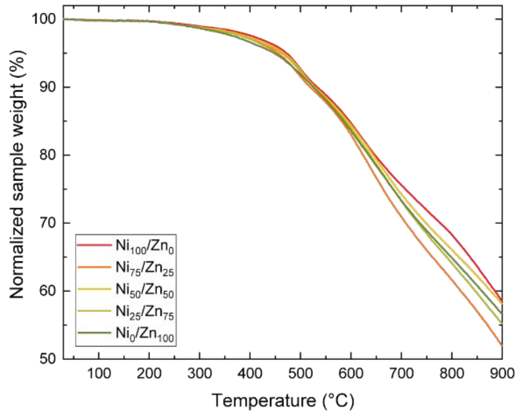

Figure S5. TGA curves of Ni/Zn-porphyrin COFs at a 10 °C/min heating rate and under a constant nitrogen flow of 20 mL/min

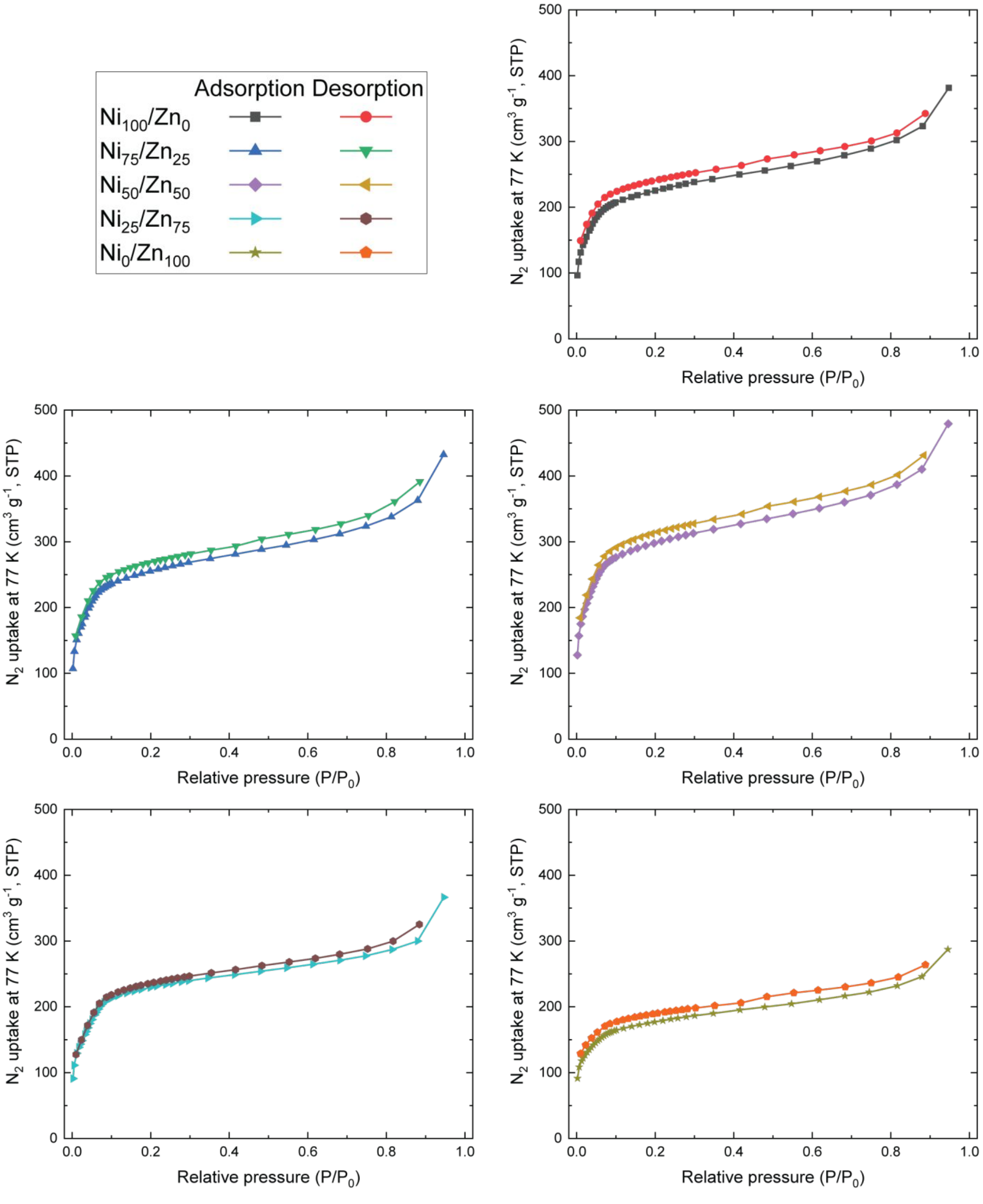

Figure S6. Nitrogen ad- and desorption isotherms of Ni/Zn-porphyrin COFs at 77 K.

A quenched solid density functional theory (QSDFT) carbon model that considers slit- and cylindrical pores, with the adsorption branch of the isotherms as experimental input values, was used to calculate the pore size distributions of Figure S7.

The small mesopore size of 2.2 nm is the expected pore size from geometry optimized crystal lattices of similar simulated COF structures.[Ref S5] The pore volume that arises from these specific pore sizes is lowest (both in absolute terms and as a percentage of the total pore volume) in the Ni_0/Zn_{100} COF, which suggests the lowest amount of eclipsed stacking of crystalline domains in this framework. Preventing monomer aggregation during COF synthesis is essential to allow the polycondensation reactions to occur effectively, which then allows the formation of a porous network. Yet, porphyrin units are known to aggregate through π-π stacking or metal-ligand interactions. Seemingly, utilising a 1:1 ratio of metalloporphyrin monomers results in a high degree of monomer dispersion and prevents self-aggregation of the pure components.

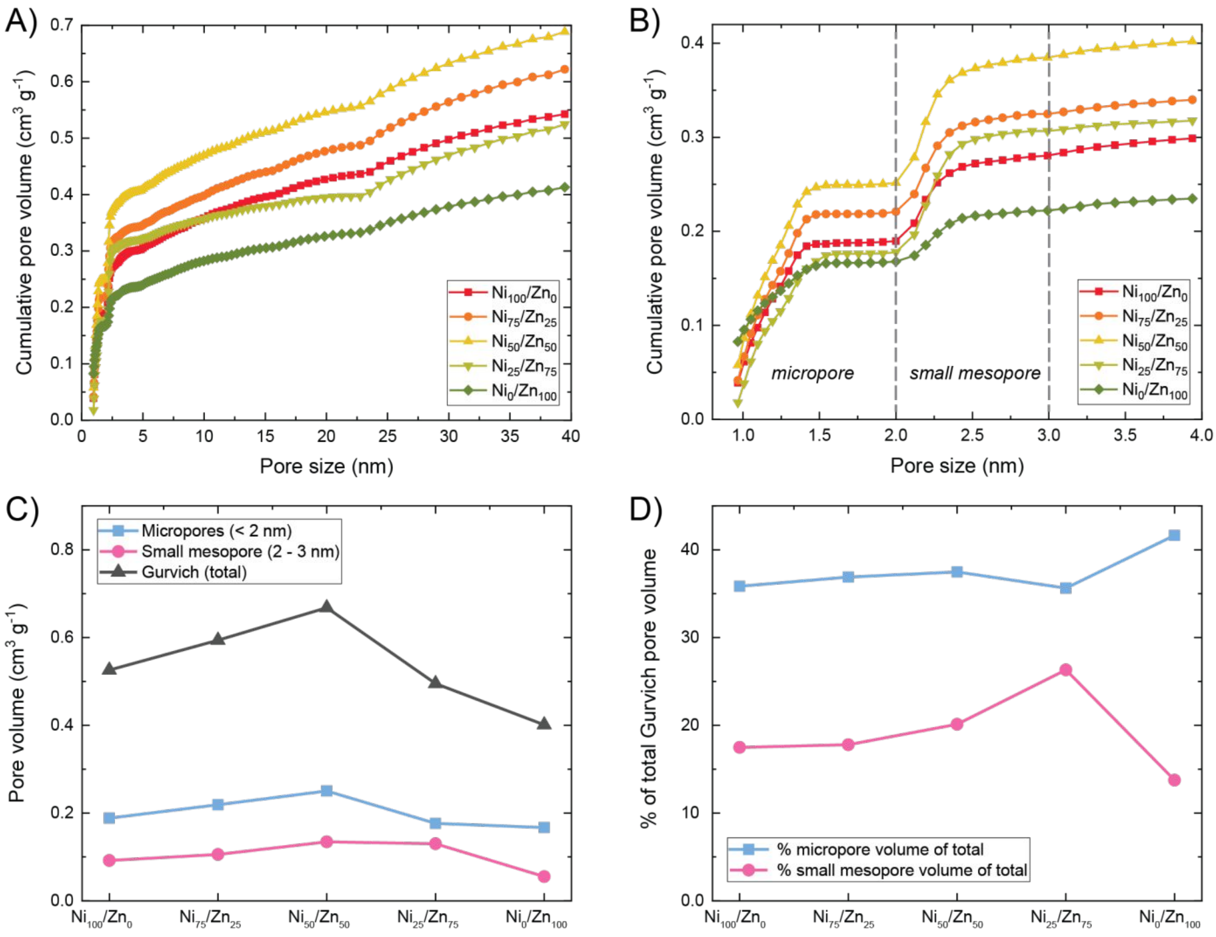

Figure S7. **A)** Cumulative pore volume of Ni/Zn-porphyrin COFs, calculated from experimental N_2 adsorption isotherm branches and based on a QSDFT carbon model with slit/cylindrical pore geometries. **B)** Zoom-in of graph A), highlighting the regions where micropores and small mesopores are most dominant. **C)** Micro- and small mesopore volumes of Ni/Zn-porphyrin COFs estimated from the dotted lines represented in graph B), as well as their Gurvich pore volumes. **D)** Micro- and small mesopore volume as a percentage of the total Gurvich pore volume.

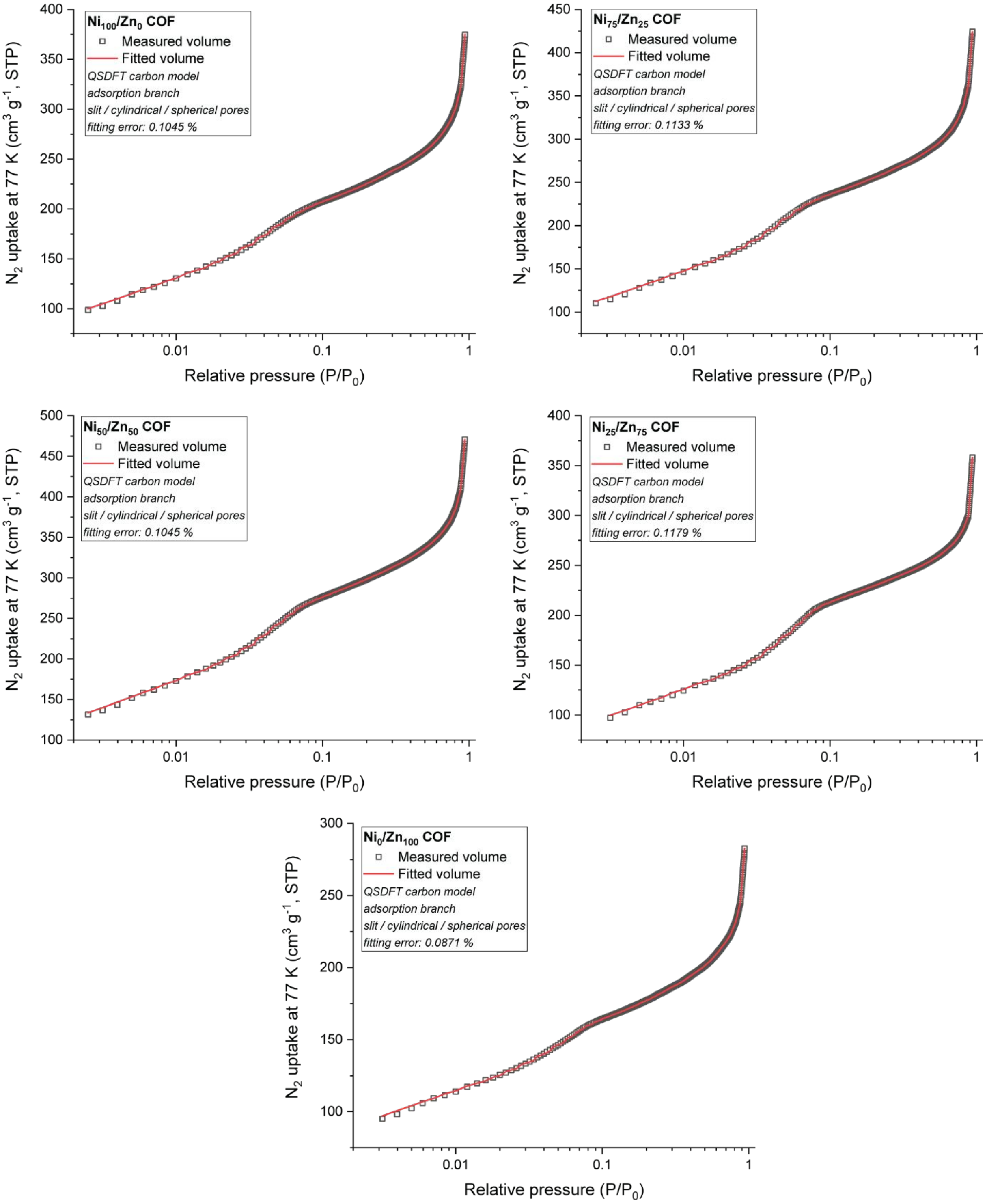

Figure S8. Fitting curves of the PSDs of Ni/Zn-porphyrin COFs, with indicated fitting errors.

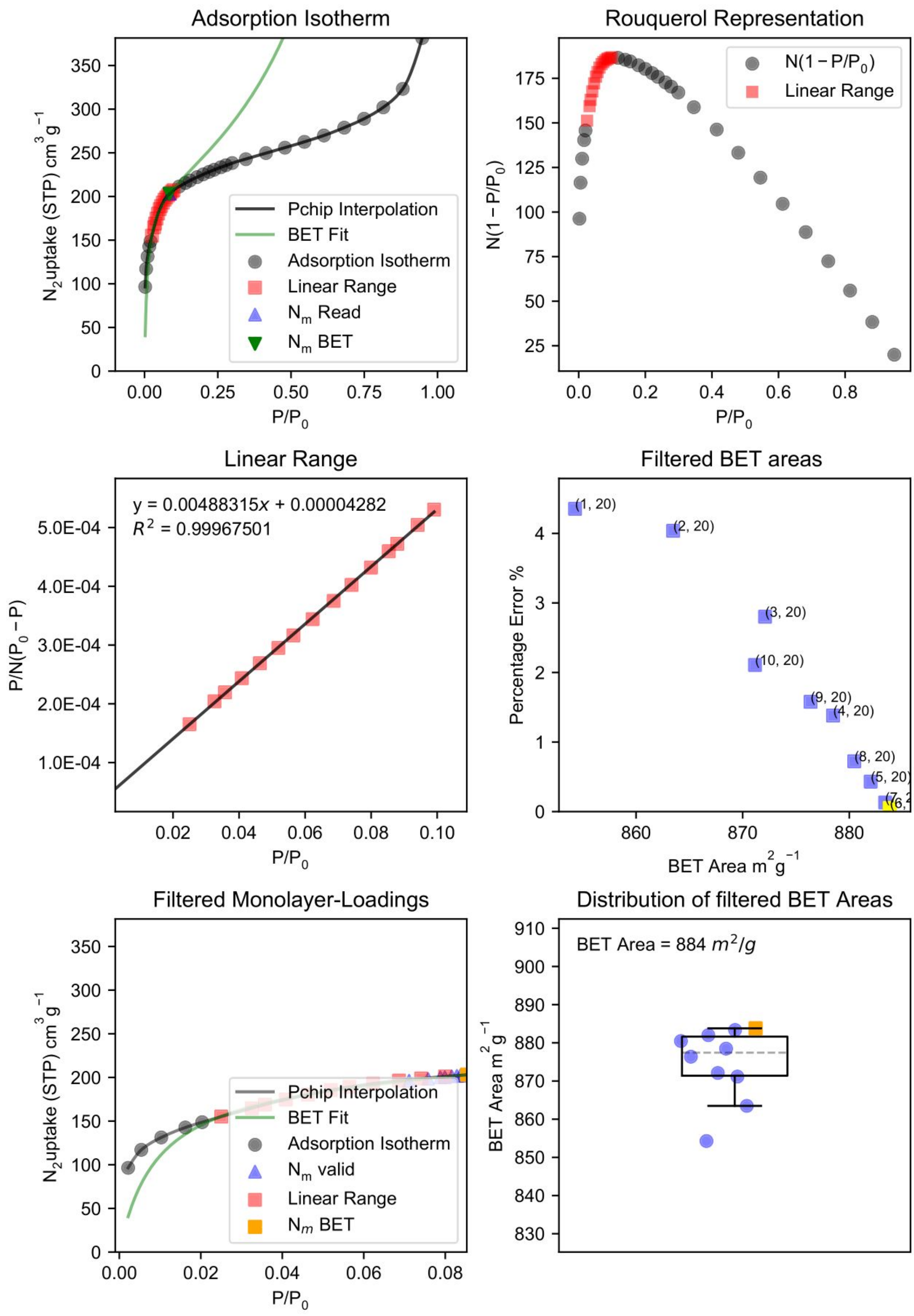

Figure S9. BETSI analysis report for the adsorption isotherm of Ni_{100}/Zn_0 COF.

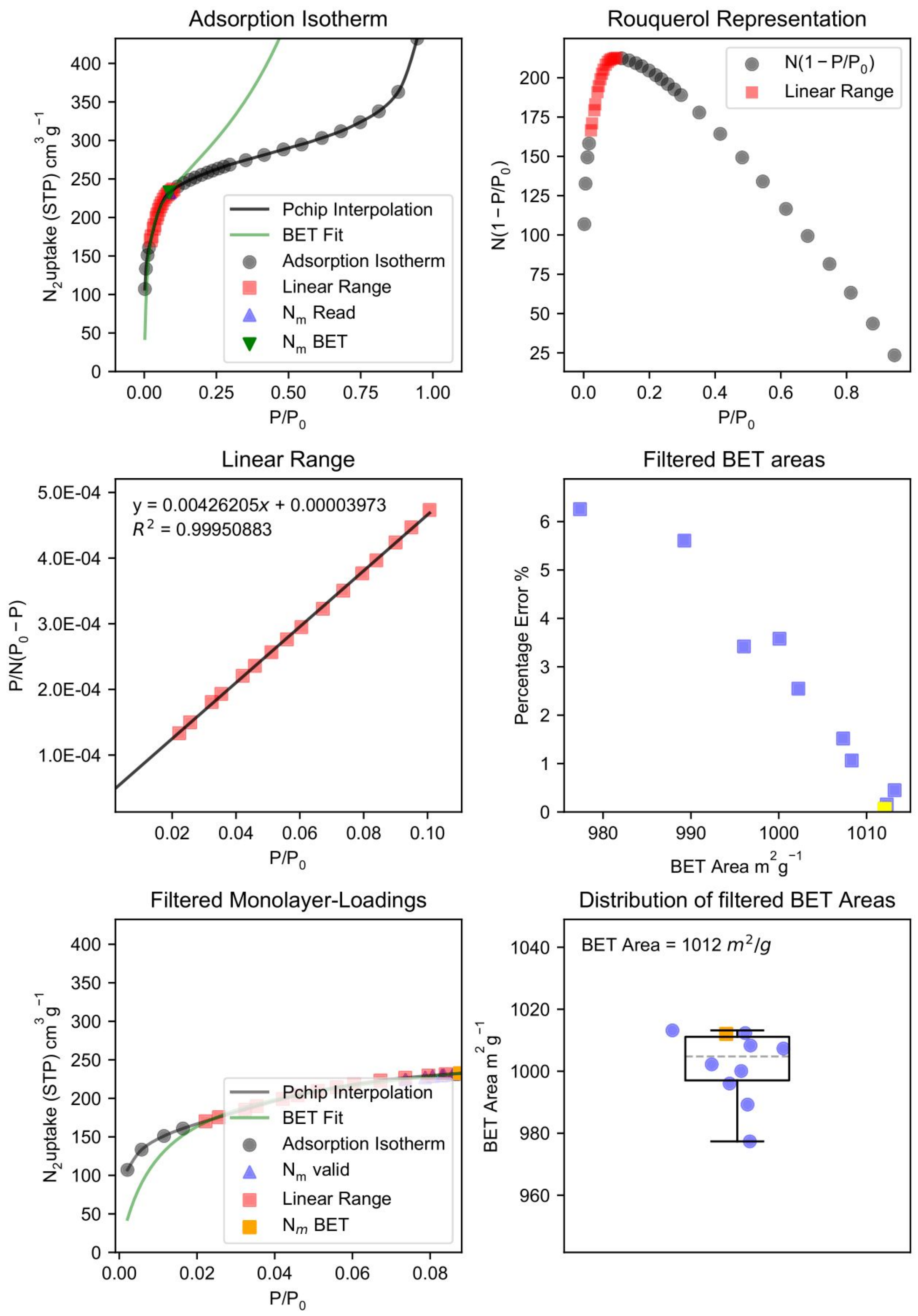

Figure S10. BETSI analysis report for the adsorption isotherm of Ni_{75}/Zn_{25} COF.

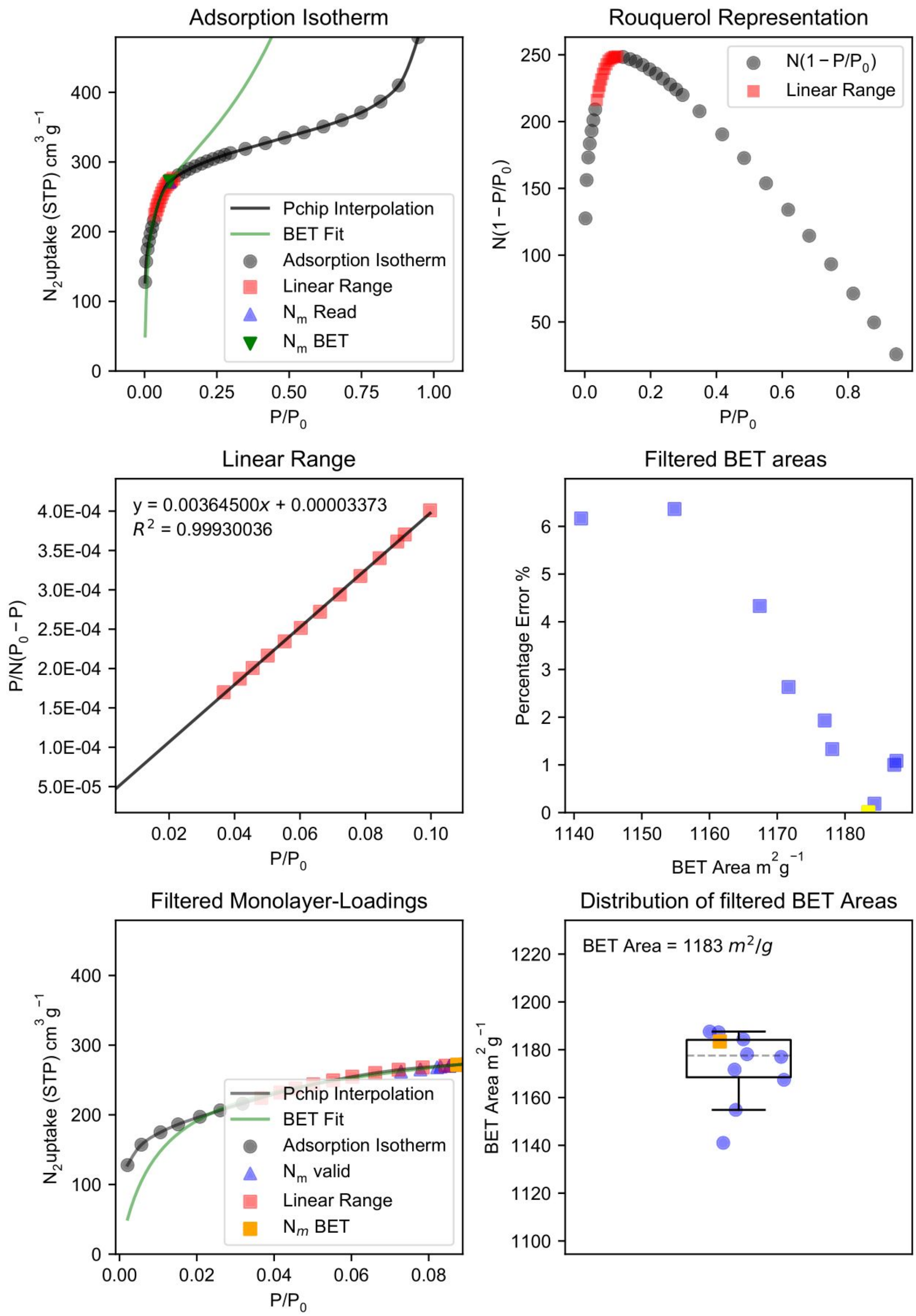

Figure S11. BETSI analysis report for the adsorption isotherm of Ni_{50}/Zn_{50} COF.

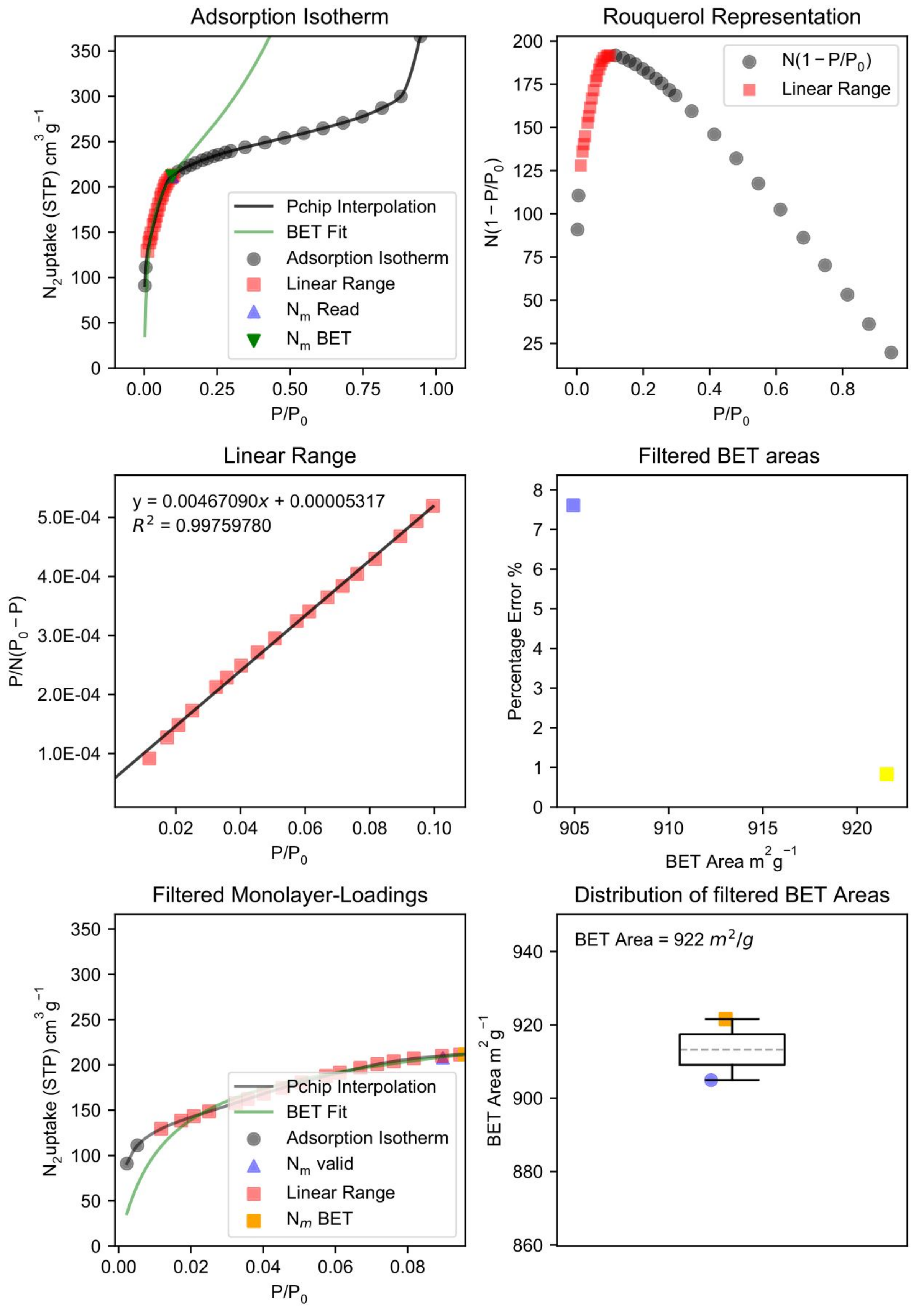

Figure S12. BETSI analysis report for the adsorption isotherm of Ni_{25}/Zn_{75} COF.

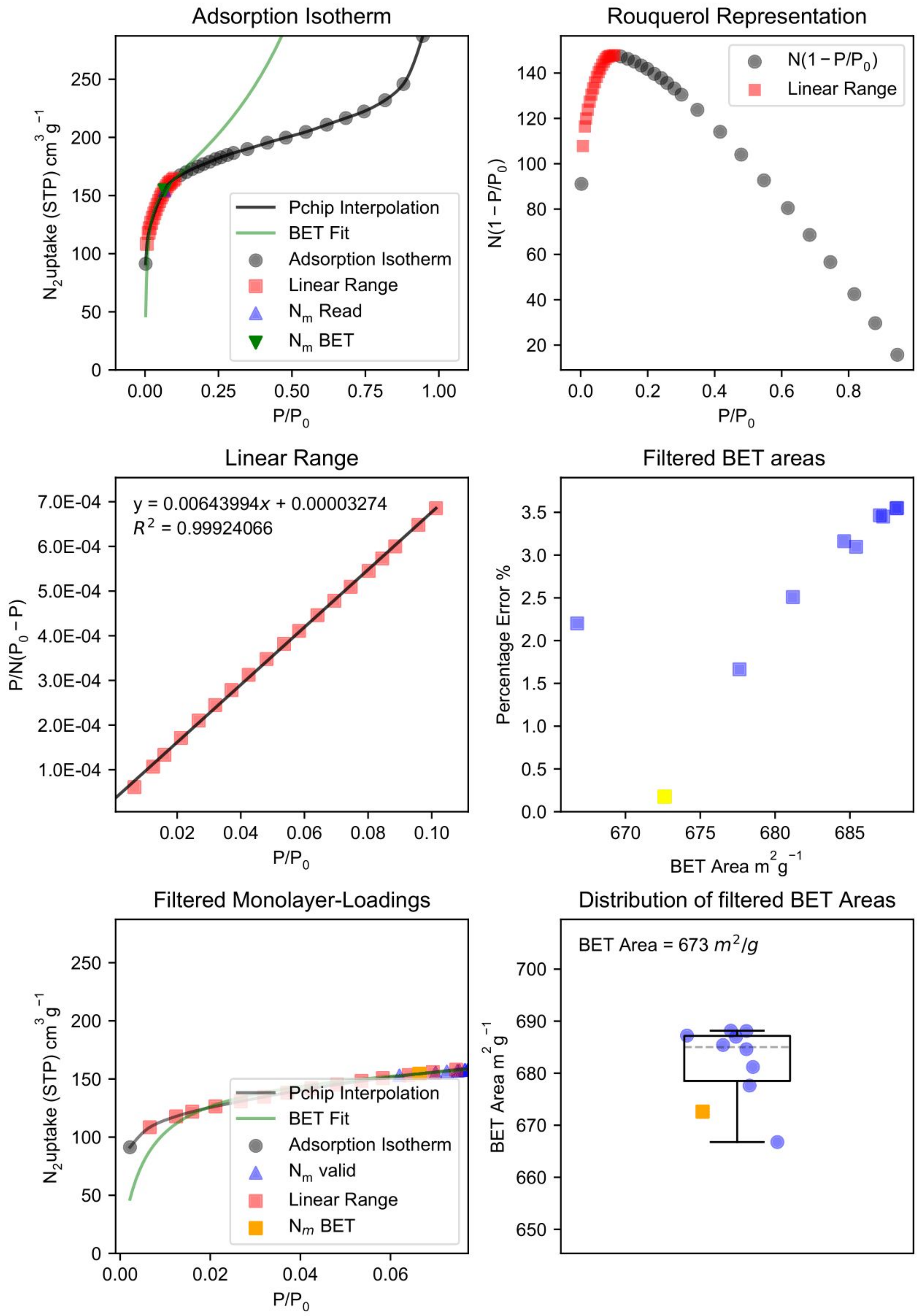

Figure S13. BETSI analysis report for the adsorption isotherm of Ni_0/Zn_{100} COF.

Focusing on the high-resolution C 1s, N 1s, O 1s, Ni 2p and Zn 2p XPS spectra (Figure S14). The C 1s and O 1s spectra of all powders are similar, revealing four different carbon species on their surfaces and one distinct oxygen state originating from the DHTA monomer's hydroxyl groups. Furthermore, two N 1s states are present for all COFs (at binding energies of ~ 398 eV and 399.5 eV) except for the Ni_0/Zn_{100} COF, which shows three N 1s states (397.2; 398.2; and 399.5 eV).

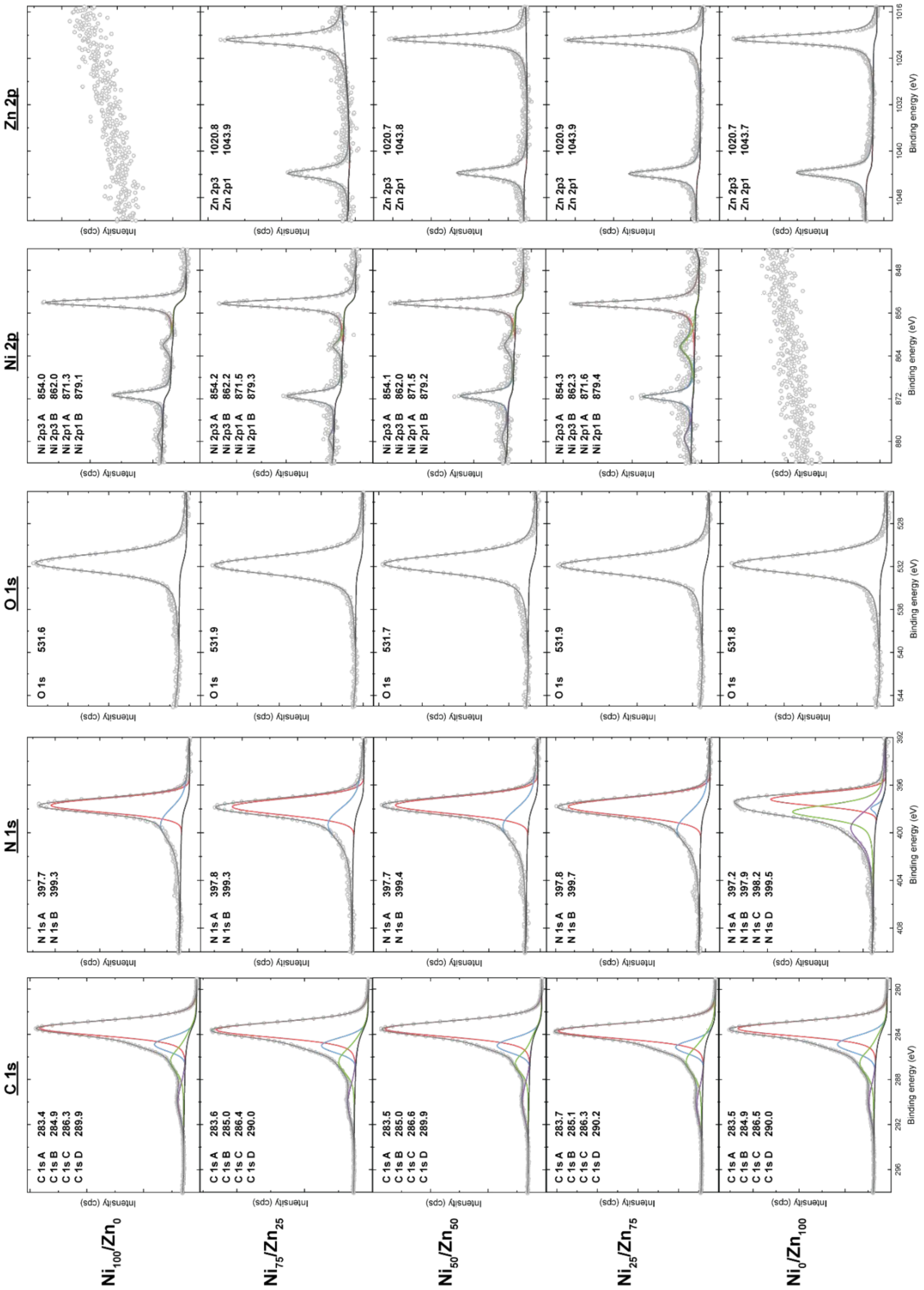

Figure S14. High-resolution N 1s, O 1s, Ni 2p, and Zn 2p XPS spectra of Ni/Zn-porphyrin COFs.

Table S1 XPS-derived atomic percentages of C, N, O, Ni, and Zn for Ni/Zn-porphyrin COFs.

	Measured						Expected		
	At. % C	At. % N	At. % O	At. % Ni	At. % Zn	Ni:Zn ratio	At. % Ni	At. % Zn	Ni:Zn ratio
**Ni_{100}/Zn_0**	80.21	10.48	8.26	1.05	0	100:0	0.92	0	100:0
**Ni_{75}/Zn_{25}**	79.31	10.58	8.73	0.9	0.49	65:35	0.69	0.23	75:25
**Ni_{50}/Zn_{50}**	79.81	10.64	7.98	0.63	0.93	40:60	0.46	0.46	50:50
**Ni_{25}/Zn_{75}**	79.44	10.42	8.57	0.45	1.12	29:71	0.23	0.69	25:75
**Ni_0/Zn_{100}**	80.47	10.4	7.62	0	1.51	0:100	0	0.92	0:100

PXRD: All investigated COFs showed a prominent peak at 3.6 ° 2θ, corresponding to a distance of 2.45 nm. Secondary peaks and shoulder peaks were observed in the region of 6 – 10 ° 2θ, but they were not very well defined, which suggests positional order to be present only on a local length scale. Although no clear trend for the pure and mixed Ni/Zn-porphyrin COFs was observed, the Ni_0/Zn_{100} COF has the most prominent shoulder peak (~ 8.3 ° 2θ). Lastly, the diffuse signal centred around 22.5 ° 2θ corresponding to an expected interplanar distance of 0.395 nm observed for all COFs suggests a significant amount of stacking of the 2D COF sheets.

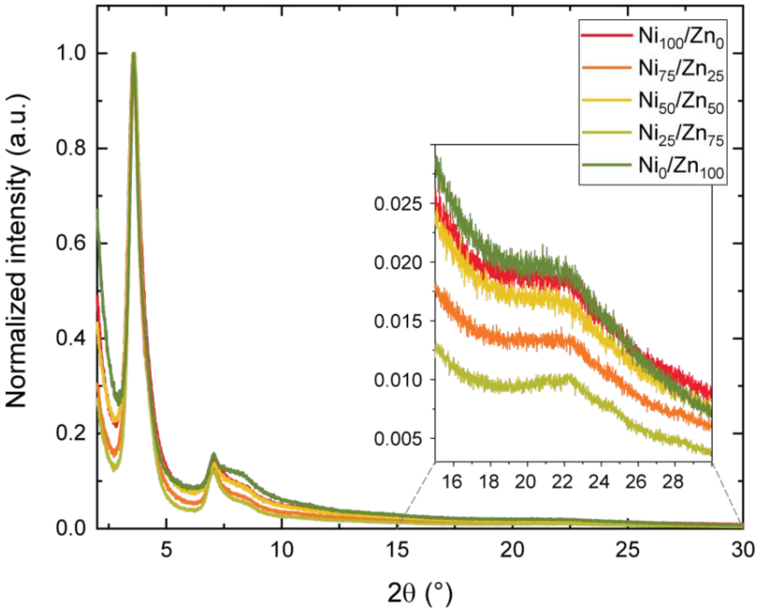

Figure S15. PXRD patterns of Ni/Zn-porphyrin COFs.

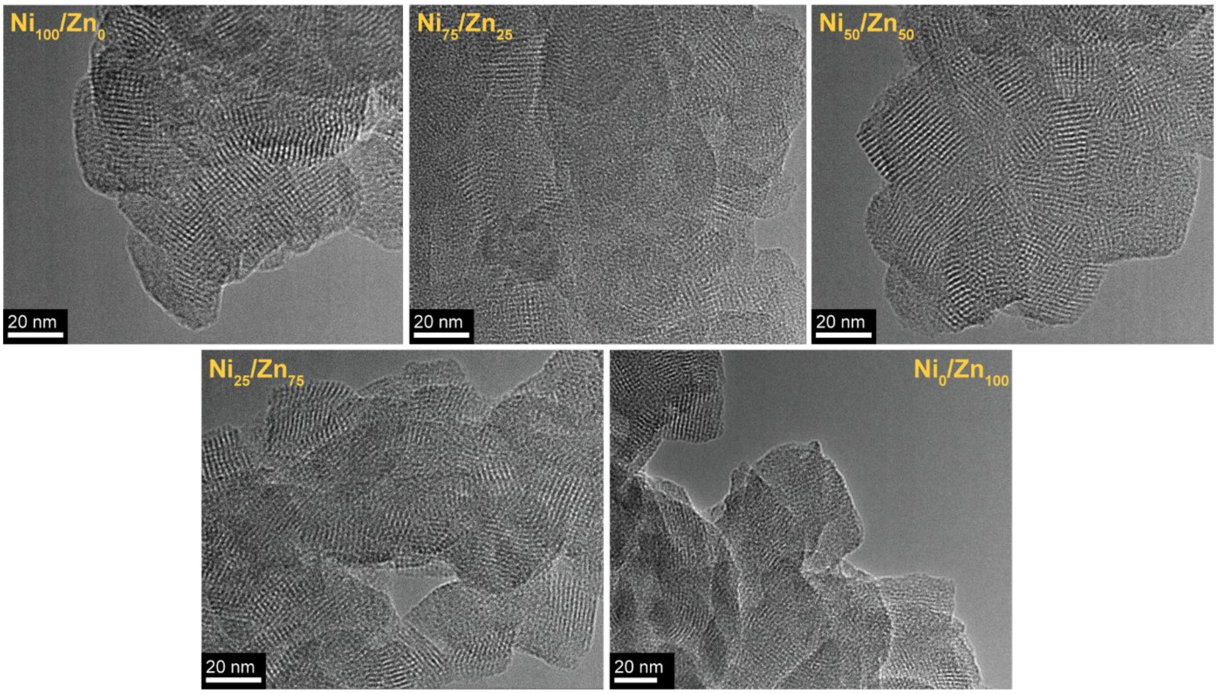

Figure S16. High-resolution TEM images of Ni/Zn-porphyrin COFs.

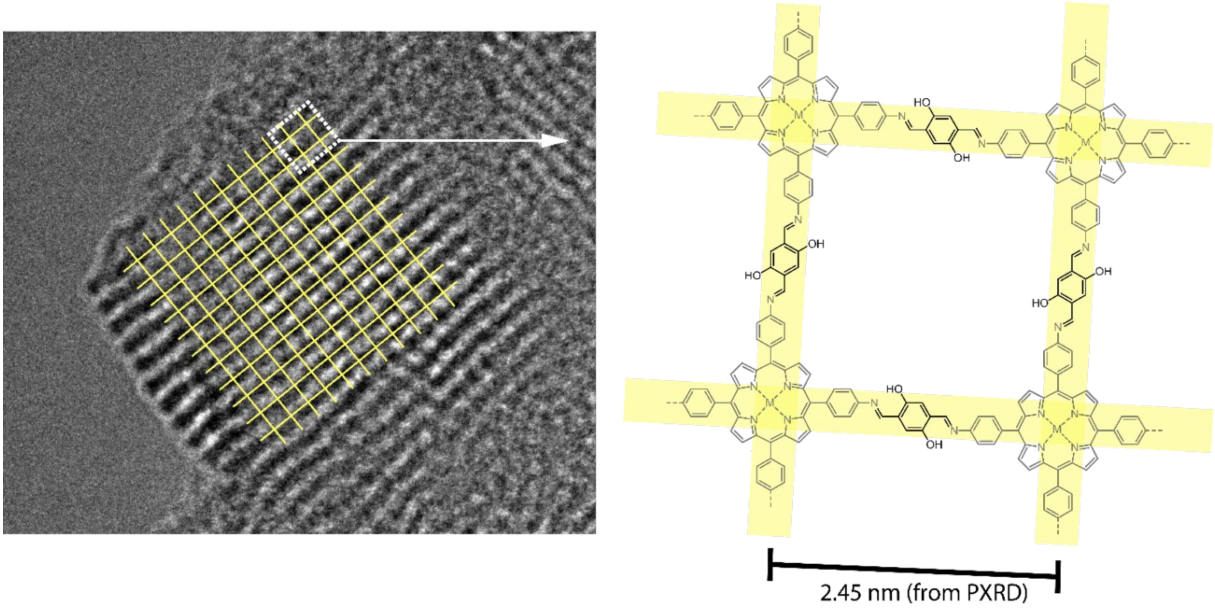

Figure S17. Correlation between the square lattice observed in HR-TEM measurements (Figure S16) and the proposed porphyrin-COF molecular structure (Figure 1a), with indicated repeating unit size based on PXRD patterns (Figure S15).

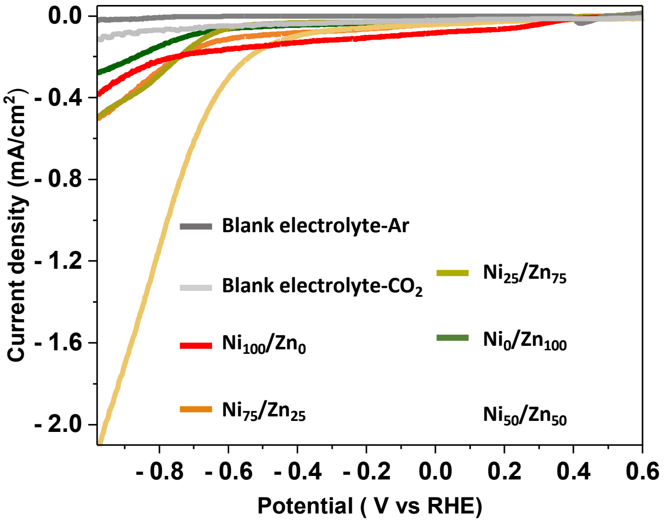

Figure S18. Linear sweep voltammetry (LSV) comparison of heterogeneous COF complexes in 0.1 M $KHCO_3$.

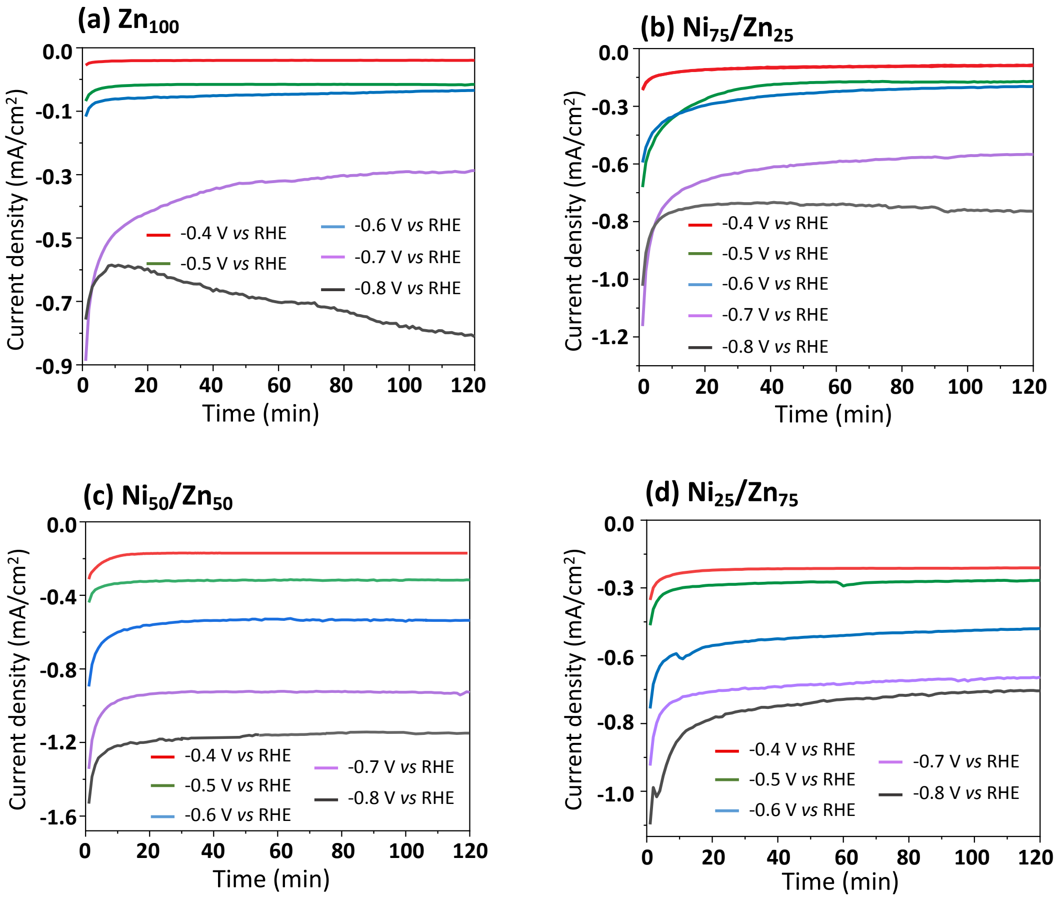

Figure S19. Chronoamperometry comparison of (a) Ni_0/Zn_{100}; (b) Ni_{75}/Zn_{25} (c) Ni_{50}/Zn_{50}; and (d) Ni_{25}/Zn_{75} at -0.4 to -0.8 V *vs* RHE in 0.1 M $KHCO_3$ H-cell.

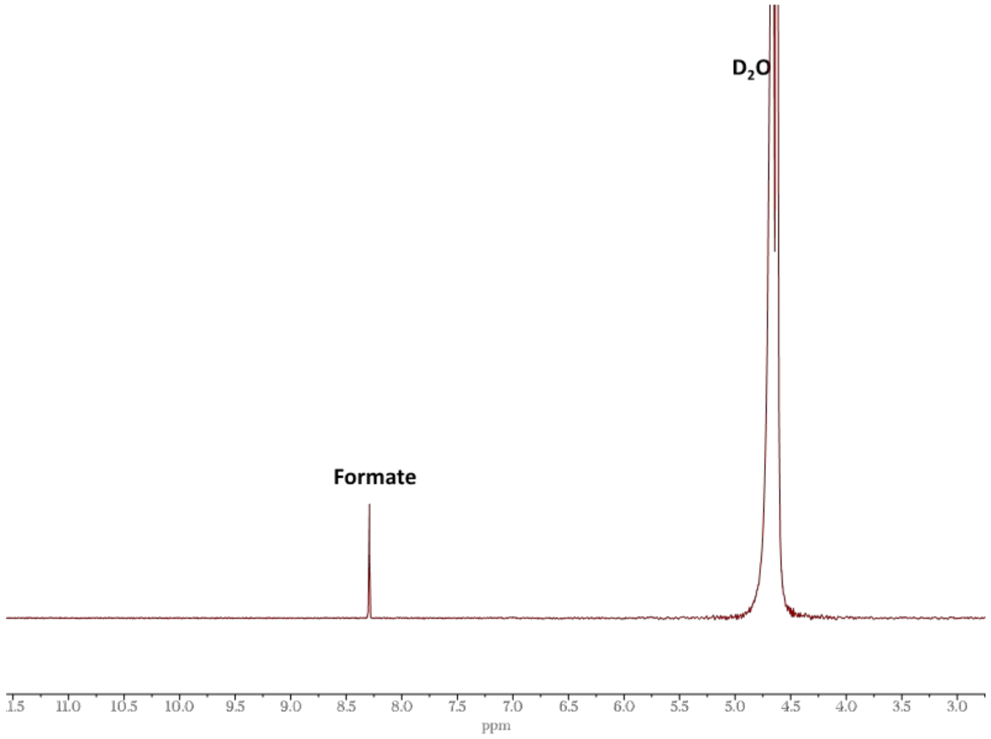

Figure S20. ^{1}H NMR spectrum example of formate obtained during CO_2 electroreduction.

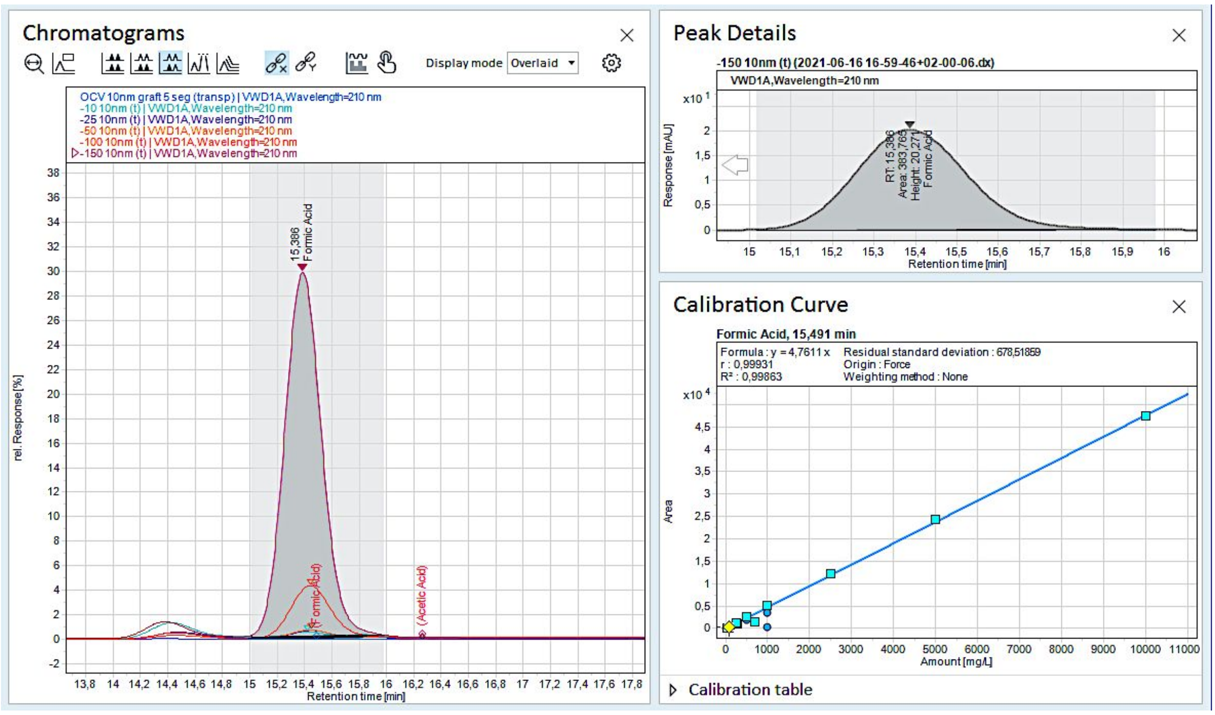

Figure S21. High-Performance Liquid Chromatography (HPLC) chromatogram example of formic acid obtained during CO_2 electroreduction.

Table S2 Product analysis of heterogeneous catalysts in a H-Cell using 0.1 M $KHCO_3$ electrolyte.

Compound	V vs RHE	j (mA/cm^2)	FE% (CO)	FE% (formate)	FE% (CH_4)	FE% (H_2)
Ni_{100}/Zn_0	-0.4	-0.02	0	0	0	100±1.5
	-0.5	-0.048	0	0	0	100±2.3
	-0.6	-0.051	4.5±2.7	0	0	95±3
	-0.7	-0.21	3±1.5	0	0	94±1.1
	-0.8	-0.36	0	0	0	100±2
Ni_0/Zn_{100}	-0.4	-0.035	21±2.8	0	0	77±1.5
	-0.5	-0.06	26±3	0	0	72±2.3
	-0.6	-0.08	46±3.1	0	0	53±3
	-0.7	-0.34	77±1.2	0	0	23±1.1
	-0.8	-0.82	41±2.5	0	0	59±2.0
Ni_{75}/Zn_{25}	-0.4	-0.11	0	0	0	100
	-0.5	-0.15	0	0	0	100
	-0.6	-0.28	27±1.1	0	0	73±3.4
	-0.7	-0.56	34±1.8	0	0	64±2
	-0.8	-0.73	10±2	0	0	80±1.5
Ni_{50}/Zn_{50}	-0.4	-0.21	38±1	0	0	66±1.5
	-0.5	-0.33	64±2.7	7±2.4	0	27±1.3
	-0.6	-0.49	69±1.2	10±2.8	0	20±2.4
	-0.7	-0.94	50±2.8	4±3.2	0	45±1.0
	-0.8	-1.1	18±3.4	0	0	82±3.1
Ni_{25}/Zn_{75}	-0.4	-0.18	0	0	0	100
	-0.5	-0.27	11±2.7	0	0	86±4.6
	-0.6	-0.46	36±2.0	8±1.4	0	55±2.0
	-0.7	-0.66	28±2.3	0	0	71±1.8
	-0.8	-0.7	15±1.5	0	0	85±3.7

Table S3 Turnover number (TON) and turnover frequency (TOF) of heterogeneous catalysts in a H-Cell using 0.1 M $KHCO_3$ electrolyte and an electrolysis time of 2 hours. TON = $n_{product}$ / $n_{catalyst}$ and TOF = TON / t. $n_{catalyst}$ was calculated based on the molecular weight of the COF-repeating unit and in case of the mixed component COFs, the molar ratio of Ni:Zn was taken into account. It is worth noting that these numbers are likely underestimated since the entire COF backbone is taken into account and not purely the active centre.

**Compound**	**Product**	**TON**	**TOF (h^{-1})**	**Potential (V vs RHE)**	**Current density (mA cm^{-2})**
Ni_{75}/Zn_{25}	CO	2494	1247	-0.7	0.49
Ni_{50}/Zn_{50}	CO, formate	6042	3021	-0.6	0.51
Ni_{25}/Zn_{75}	CO, formate	3371	1685	-0.6	0.51
Ni_{0}/Zn_{100}	CO	3824	1912	-0.7	0.33

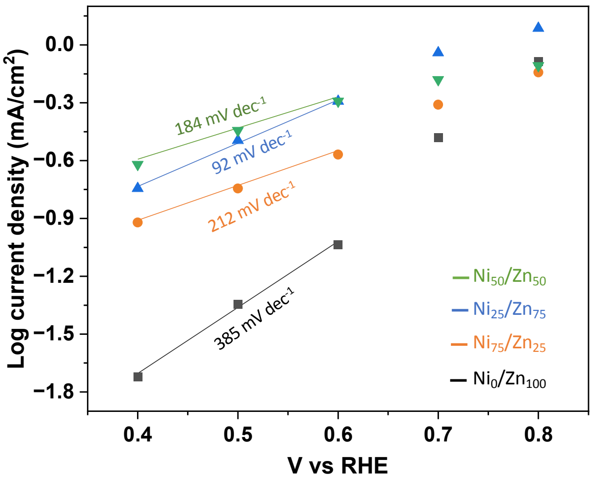

Figure S22. Tafel slopes for the current density of the synthesized catalysts at -0.4, -0.5, -0.6, -0.7, and -0.8, V vs RHE in 0.1 M $KHCO_3$.

For experiments with higher current densities, a membrane electrode assembly (MEA) electrolyzer consisting of an anode chamber (Ni-foam anode, Recemat BV) with a liquid phase anolyte (0.5 M KOH) and a cathode chamber (COF on GDE) with a gas phase inlet was employed (schematic shown at Figure S22). The gas products were collected from the cathode and characterized using gas chromatography (GC), while the liquid products were collected from the anolyte through a Teflon tube immersed in the anolyte solution, facilitating continuous liquid sample collection throughout the process. and characterized using both ^{1}HNMR and high-performance liquid chromatography (HPLC).

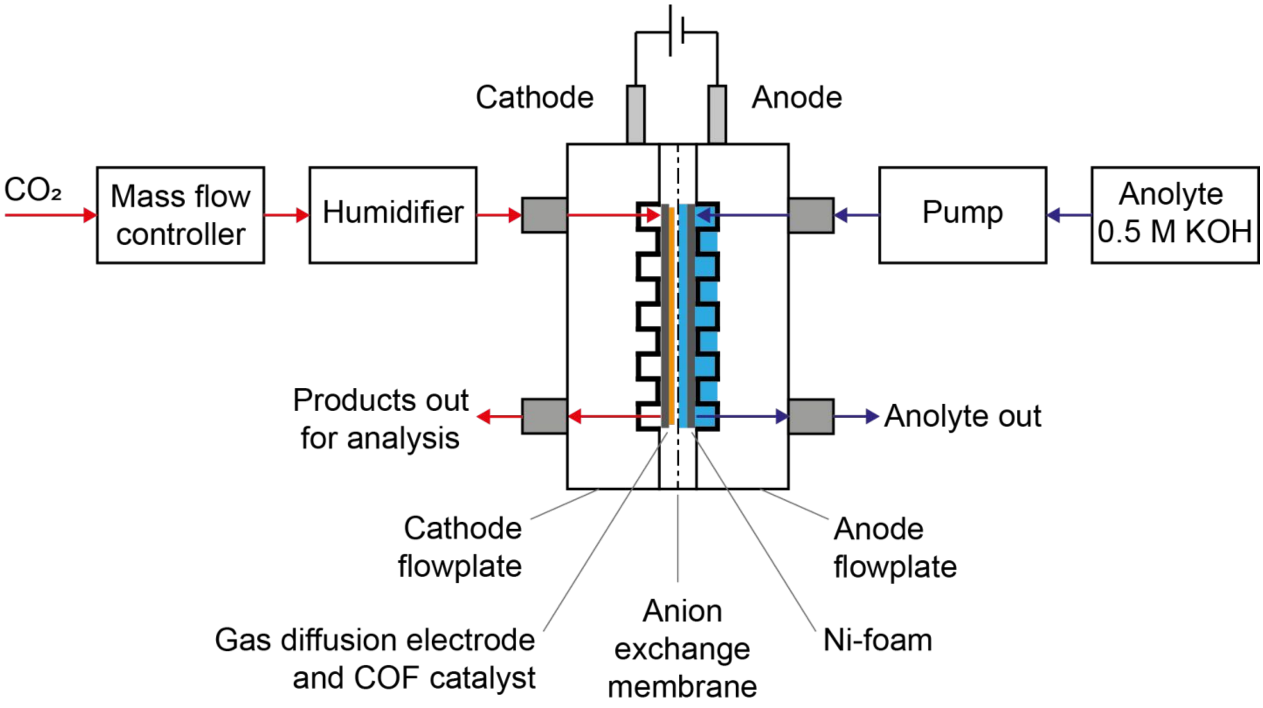

Figure S23. Schematic of MEA reactor.

Table S4. Product analysis of heterogeneous catalysts in a MEA Cell using 0.5 M KOH electrolyte.

Compound	V vs RHE	*j* (mA/cm^2)	FE% (CO)	FE% (formate)	FE% (CH_4)	FE% (H_2)
Ni_{100}/Zn_0	-2,76	25	6±3.7	0	0	93±1.5
	-2,83	50	8±2.5	0	0	91±2.3
	-2,88	100	7±4.8	3±2.8	0	86±3
	-3,06	150	4±1.2	0	0	90±1.1
Ni_0/Zn_{100}	-2,7	25	73±1.7	0	0	25±1.7
	-2,86	50	78±1.1	3±0.8	0	16±1.0
	-3,13	100	79±0.9	9±1.2	0	10±0.6
	-3,19	150	44±2.5	4±2.8	0	43±0.7
Ni_{75}/Zn_{25}	-2,68	25	41±1.4	0	0	55±2.6
	-2,71	50	40± 1.0	0	0	59±1.1
	-2,91	100	23±0.8	0	0	74±1.8
	-3,14	150	12±0.8	0	0	76±1.4
Ni_{50}/Zn_{50}	-2,58	25	54±1.3	31±1.4	0	14±0.8
	-2,7	50	43±0.8	34±0.5	5±1.0	17±1.5
	-2,88	100	37±1.4	40± 2.1	11±2.3	12±1
	-3	150	24±0.7	43±1.0	14±1.1	16±2.2
Ni_{25}/Zn_{75}	-2,86	25	60±1.9	4±1.0	0	33±1.8
	-3,14	50	64±2.3	7±1.2	0	28±1.9
	-3,3	100	52±1.5	11±1.7	0	35±0.8
	-3,75	150	36±3.8	19±4.1	0	42±1.5

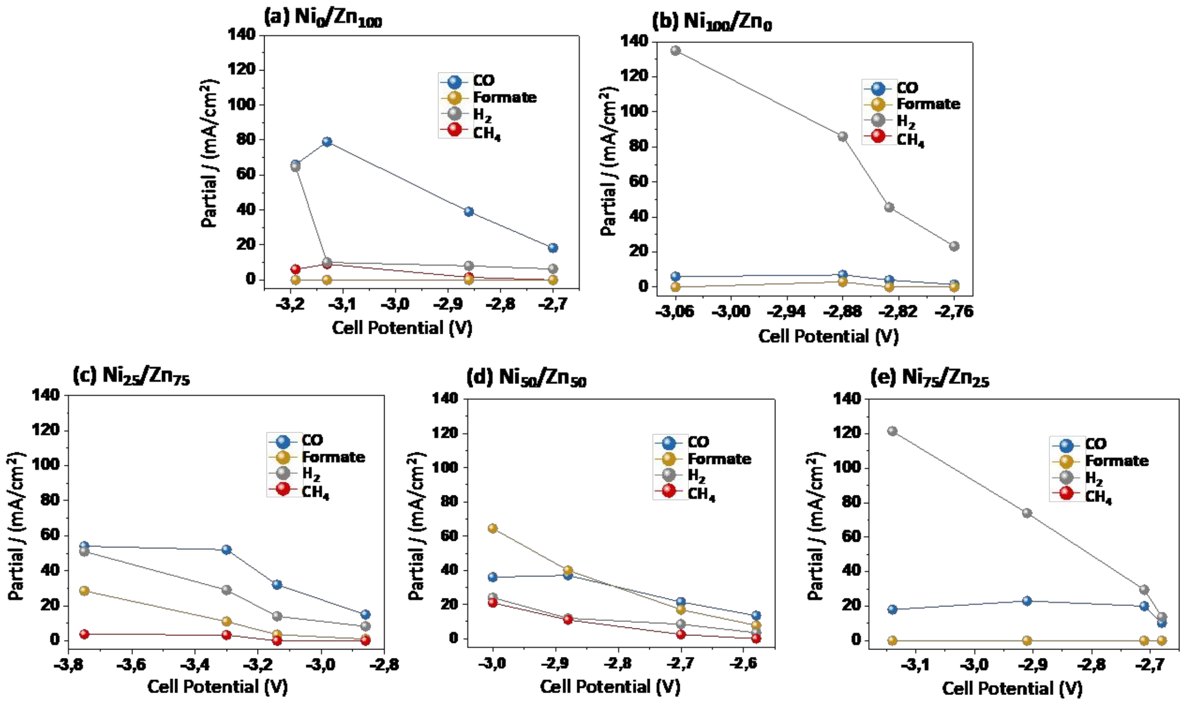

Figure S24. Partial current density comparison of (a) Ni_0/Zn_{100}; (b) Ni_{100}/Zn_0; (c) Ni_{25}/Zn_{75}; (d) Ni_{50}/Zn_{50}; and (e) Ni_{75}/Zn_{25} at -2.7 to -3.8 V in 0.5 M KOH.

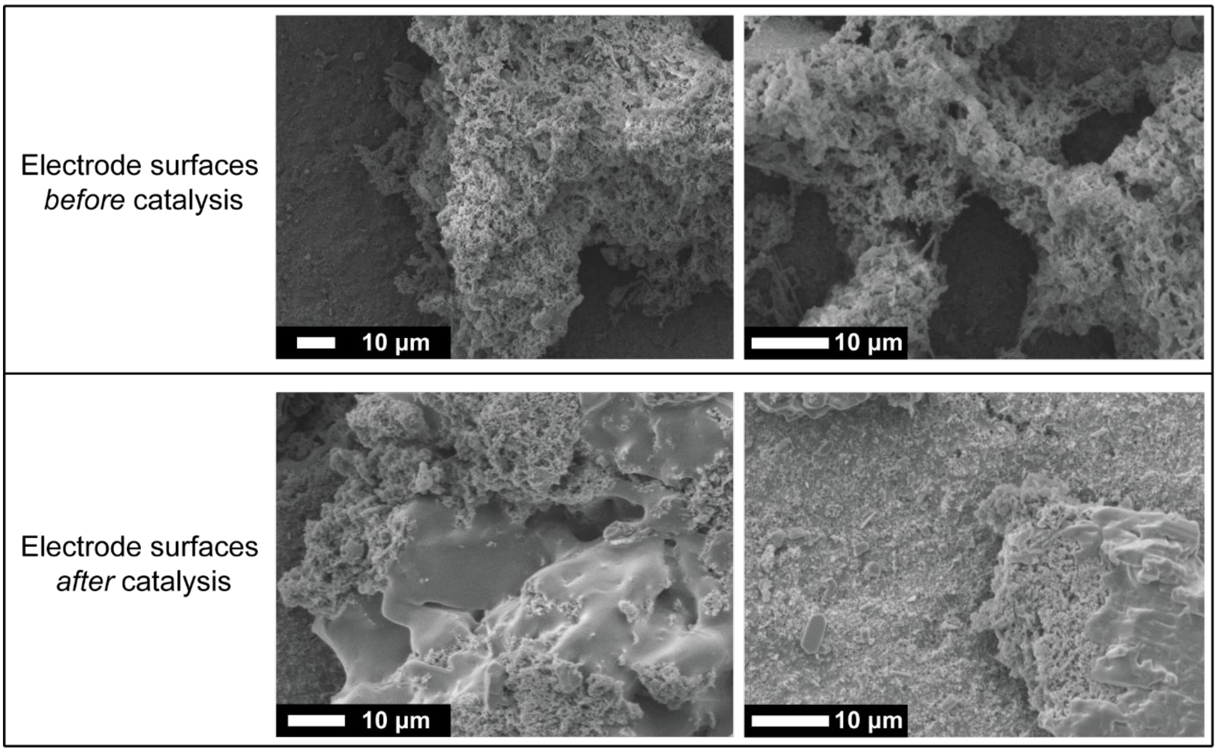

Figure S25. Scanning electron microscopy images of representative Ni_{50}/Zn_{50}-COF particles embedded onto the GDE surface: before (top) and after (bottom) CO_2 electroreduction experiments.

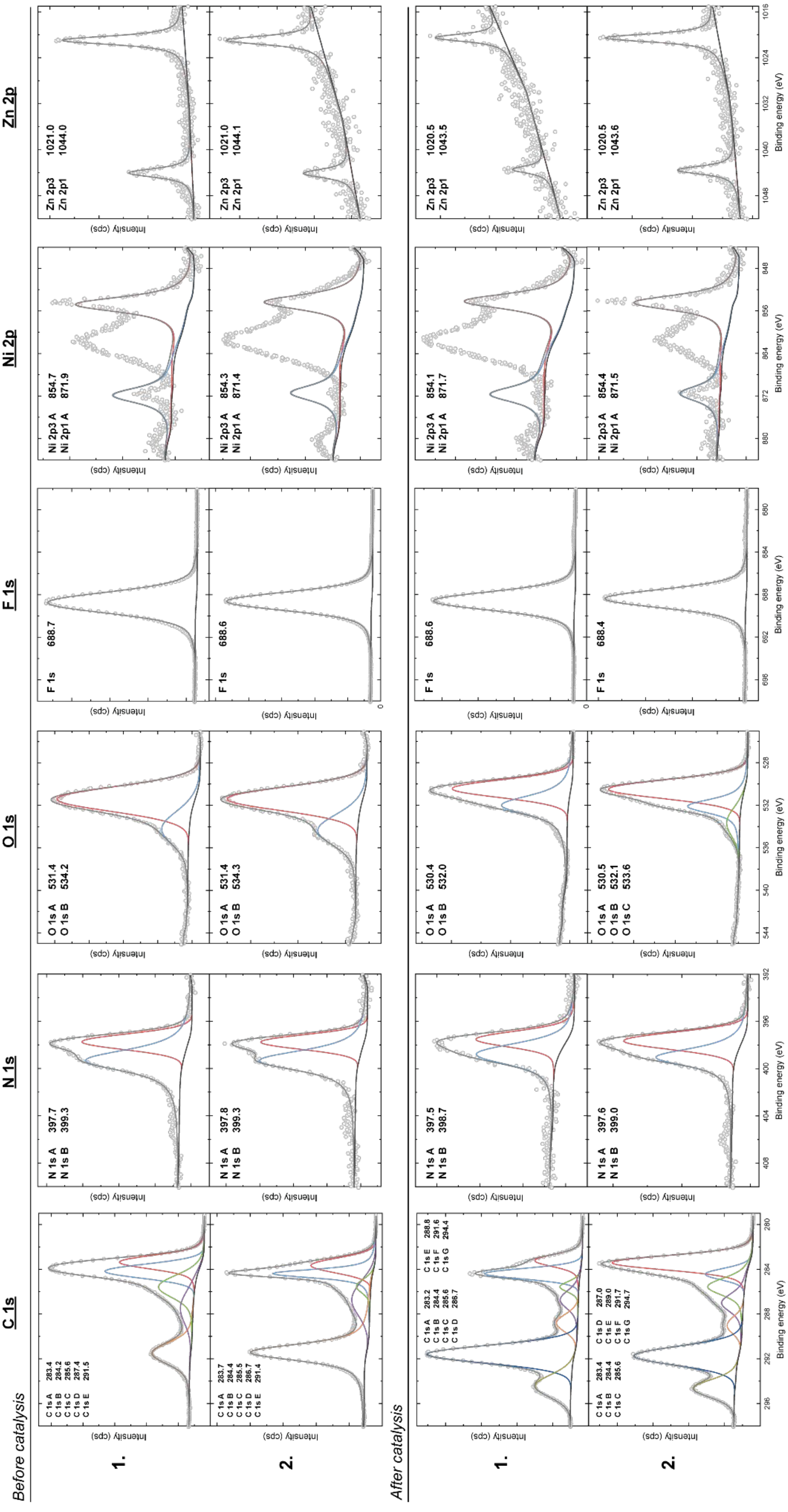

Before catalysis
After catalysis
C 1s
N 1s
O 1s
F 1s
Ni 2p
Zn 2p
1.
2.
C 1s A 283.4
C 1s B 284.2
C 1s C 285.6
C 1s D 287.4
C 1s E 291.5
C 1s A 283.7
C 1s B 284.4
C 1s C 285.5
C 1s D 286.7
C 1s E 291.4
C 1s A 283.2
C 1s B 284.4
C 1s C 285.6
C 1s D 286.7
C 1s E 288.8
C 1s F 291.6
C 1s G 294.4
C 1s A 283.4
C 1s B 284.4
C 1s C 285.6
C 1s D 287.0
C 1s E 289.0
C 1s F 291.7
C 1s G 294.7
N 1s A 397.7
N 1s B 399.3
N 1s A 397.8
N 1s B 399.3
N 1s A 397.5
N 1s B 398.7
N 1s A 397.6
N 1s B 399.0
O 1s A 531.4
O 1s B 534.2
O 1s A 531.4
O 1s B 534.3
O 1s A 530.4
O 1s B 532.0
O 1s A 530.5
O 1s B 532.1
O 1s C 533.6
F 1s 688.7
F 1s 688.6
F 1s 688.6
F 1s 688.4
Ni 2p3 A 854.7
Ni 2p1 A 871.9
Ni 2p3 A 854.3
Ni 2p1 A 871.4
Ni 2p3 A 854.1
Ni 2p1 A 871.7
Ni 2p3 A 854.4
Ni 2p1 A 871.5
Zn 2p3 1021.0
Zn 2p1 1044.0
Zn 2p3 1021.0
Zn 2p1 1044.1
Zn 2p3 1020.5
Zn 2p1 1043.5
Zn 2p3 1020.5
Zn 2p1 1043.6
Intensity (cps)
Binding energy (eV)

Figure S26. High-resolution N 1s, O 1s, Ni 2p, and Zn 2p XPS spectra of GDE surfaces with embedded Ni_{50}/Zn_{50}-COF particles, before (top 1. and 2.) and after (bottom 1. and 2.) CO_2 electroreduction experiments. "1." and "2." represent measurements at two different locations on the GDE surface.

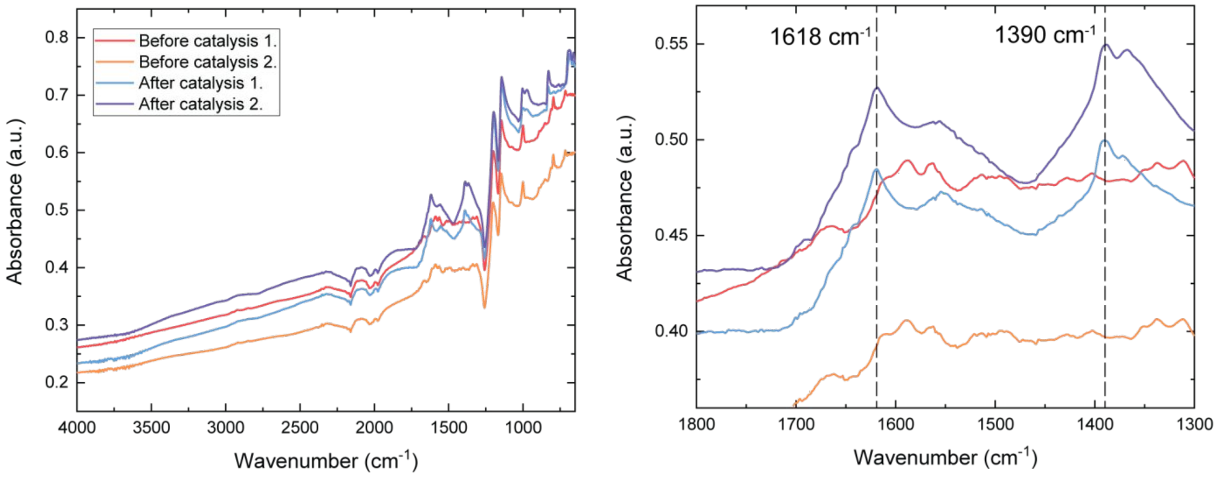

Figure S27. ATR FT-IR spectra of GDE surfaces with embedded Ni_{50}/Zn_{50}-COF particles; before and after CO_2 electroreduction experiments. "1." and "2." represent measurements at two different locations on the GDE surface.

Speculation for CO_2 to CH_4 mechanism using the Ni_{50}/Zn_{50} COF catalyst (Figure S26).

Mechanism (a) illustrates the stepwise desorption of CO from zinc, followed by adsorption onto nickel, where it is converted into CH_4. This mechanism relies on differences in adsorption affinities of metal−CO complexes, of which it is known that the interaction energy of Ni−CO (-53 kJ·mol^{-1}) is greater than the one of Zn−CO (-27 kJ·mol^{-1}).[Ref S6] The proximity of Ni- and Zn-active sites in the Ni_{50}/Zn_{50} COF (average of ~ 0.5 – 2 nm based on Figure 2b) allows easily desorbed CO from Zn sites to strongly adsorb onto Ni sites where it can be reduced further to CH_4. Chen and coworkers[Ref S7] have recently proposed a similar mechanism in the electroreduction of CO_2 using a Cu-phthalocyanine-based MOF, where the formation of C_2H_4 relies on the preferred desorption of intermediate products from one of the two neighboring active sites. Mechanism (a) can still be valid in the case of the Ni_{50} + Zn_{50} physical mixture, which explains the trace amounts of CH_4 that this catalyst was able to form (Figure 5). In the case of Ni_{50} + Zn_{50}, however, the Ni-Zn average distance is more likely to approach the COF particle size range (10 – 100 μm, as estimated from Figure S24) and only likely to be in the nanometer range at the interface of the separate COF particles. Detecting CH_4 at a higher current density in the MEA cell, and not at the low current density in the case of H-cell, highlights the

facilitation of electron transfer in high current densities, hence increasing the possibility of CH_4 formation.

The supply of electrons and protons from Ni active sites to the Zn−CO site is proposed to be the dominant pathway in mechanism (b). Ni_{100}/Zn_0 catalysts have been shown to produce a large amount of H_2 through water electrolysis. Abundant nickel hydride complexes are generated, allowing the possibility of hydrogen atom transfer towards the Zn−CO site, which are required for the conversion into CH_4. Since the Ni- and Zn-TAPP phenyl rings are oriented (close to) perpendicular with respect to the porphyrin ring, it is unlikely that proton-coupled electron transfer occurs within one COF sheet because of the poor conjugation. However, efficient charge transfer is known to occur in stacked porphyrin units,[Ref S8] and has been proven in stacked porphyrin-based COF sheets.[Ref S9]

Mechanism (c) assumes that Ni-porphyrin units solely provide a structural benefit for Zn active sites to be able to convert CO into CH_4. The porosity measurements of the powders showed that implementation of Ni-porphyrin units in Zn-porphyrin-based COFs creates a larger micro- and mesoporosity, resulting in more accessible zinc active sites. A similar phenomenon has been observed by Yaghi *et al.*, where inert Cu-porphyrins provide a structural benefit to catalytically active Co-porphyrins.[Ref S10] Xin and coworkers have proven that – given sufficient electrical conductance, microporosity, and stability – single-site Zn catalysts are able to produce CH_4 in high (FE 85 %) efficiencies.[Ref S11]

Lastly, this discussion has largely focussed on the formation of CH_4 on the Ni_{50}/Zn_{50} catalyst, as this is, in our view, the most unexpected yet most promising result. In addition, the relatively large quantities of formate (FE: 43% at 150 mA/cm^2) on the Ni_{50}/Zn_{50} catalyst are also worth discussing. A single definitive reaction pathway of CO_2 towards formate is not available, but a consensus is that formate formation is a competing reaction with CO formation.[Ref S12,Ref S13] Cheng et al.[Ref S12] investigated competing CO and formate reaction mechanisms on a Cu(100) surface through quantum mechanics calculations with an explicit description of water. Here, they claim that direct reduction of CO_2 by metal hydrides leads to $HCOO^-$ formation. Comparing the Ni_{50}/Zn_{50} catalyst to the catalysts with higher nickel content, H_2 formation is inhibited to an even larger degree than expected based on the linear average. It is possible that the hydrides that are generated on the Ni-sites during water splitting are made available for subsequent formate formation reactions. Therefore, it is reasonable to suggest a similar pathway as mechanism (b) for formate formation on Zn-sites through hydride transfer from the neighbouring Ni-sites.

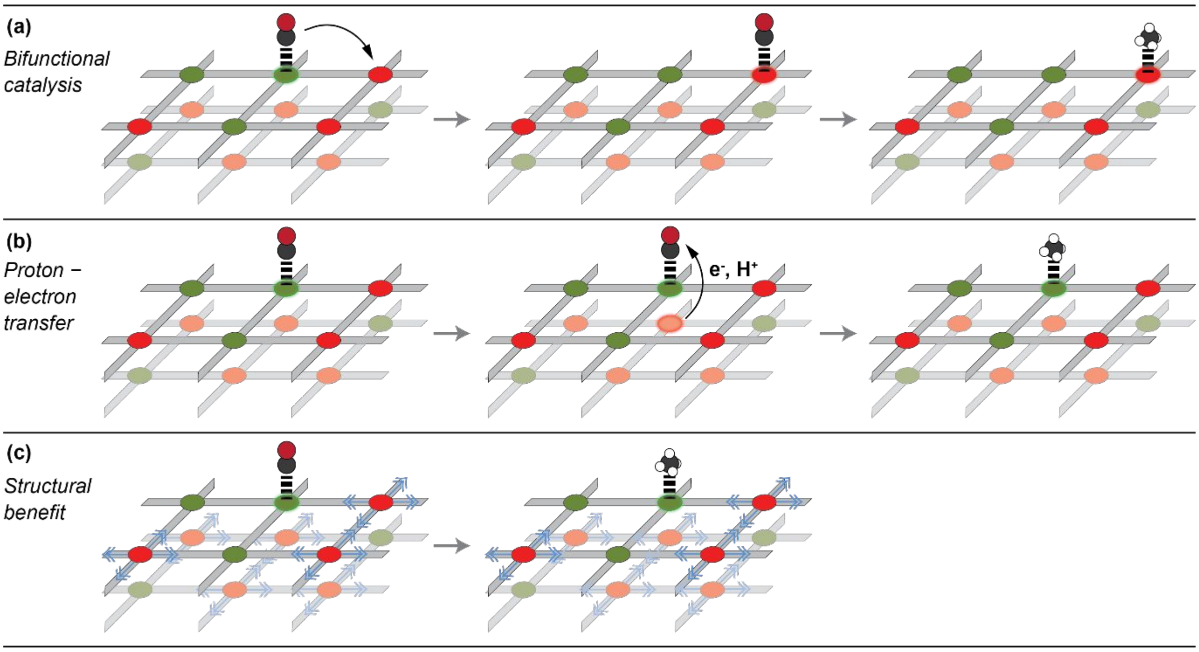

Figure S28. Schematic overview of possible simplified mechanisms behind the electrochemical conversion of CO into CH_4 using Ni_{50}/Zn_{50} COF catalysts, where nickel-sites are indicated in red and zinc-sites in green. (a): stepwise Zn−CO desorption, Ni−CO adsorption, and CO to CH_4 conversion on Ni active sites. (b): Zn active sites producing CH_4, supported by e-/H+ transfer through interlayer interactions. (c): Ni-porphyrin units providing a structural benefit.

Table S5. DFT derived energy values of Ni(II)- and Zn(II)-TPP complexes with and without the presence of CO.

**Compound**	**U (eV)**	**H (eV)**	**S*T (eV)**	**G (eV)**	**Ground state**	**Binding energy (eV)**	**Binding energy (kJ/mol)**
**Ni(II)TPP (LS)**	-92973.258	-92973.233	2.681979802	-92975.915	True		
**Ni(II)TPP (HS)**	-92973.041	-92973.016	2.740709895	-92975.756	False		
**Zn(II)TPP**	-100348.274	-100348.24	2.728370613	-100350.976	True		
**Ni(II)TPP-CO (LS)**	-96050.556	-96050.531	2.857931164	-96053.389	False	-0.24978242	-24.10033091
**Ni(II)TPP-CO (HS)**	-96050.657	-96050.631	2.880191994	-96053.512	True	-0.350596848	-33.82744084
**Zn(II)TPP-CO**	-103425.624	-103425.598	2.930925472	-103428.529	True	-0.301774426	-29.116795
**CO**	-3077.0739	-3077.0482	0.610290748	-3077.658	True		

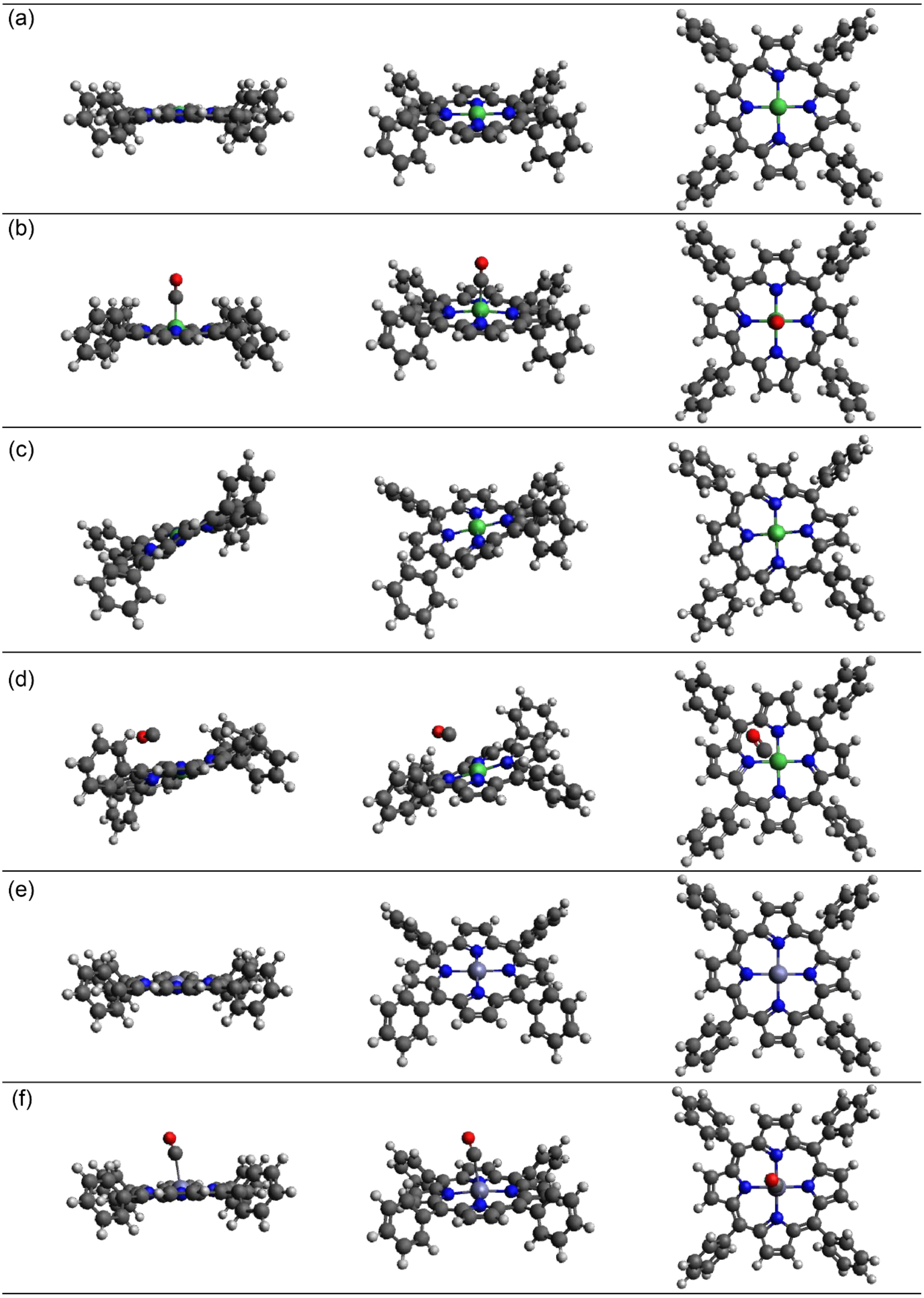

Figure S29. DFT optimized structures of (a) Ni(II)TPP-HS, (b) Ni(II)TPP-HS-CO, (c) Ni(II)TPP-LS, (d) Ni(II)TPP-LS-CO, (e) Zn(II)TPP, and (f) Zn(II)TPP-CO.

References supporting information

[Ref S1] Thomas, D. W.; Martell, A. E. Metal Chelates of Tetraphenylporphine and of Some p-Substituted Derivatives. *J. Am. Chem. Soc.* **1959**, *81*, 5111–5119.

[Ref S2] Ogoshi, H.; Saito, Y.; Nakamoto, K. Infrared Spectra and Normal Coordinate Analysis of Metalloporphins. *J. Chem. Phys.* **1972**, *57*, 4194–4202.

[Ref S3] Wölfle, T.; Görling, A.; Hieringer, W. Conformational flexibility of metalloporphyrins studied by density-functional calculations. *Phys. Chem. Chem. Phys.* **2008**, *10*, 5739–5742.

[Ref S4] Olsson, S.; Dahlstrand, C.; Gogoll, A. Design of oxophilic metalloporphyrins: an experimental and DFT study of methanol binding. *Dalton Trans.* **2018**, *47*, 11572–11585.

[Ref S5] Qian, Y.; Li, D.; Han, Y.; Jiang, H.-L. Photocatalytic Molecular Oxygen Activation by Regulating Excitonic Effects in Covalent Organic Frameworks. *J. Am. Chem. Soc.* **2020**, *142*, 20763−20771.

[Ref S6] Bloch, E. D.; Hudson, M. R.; Mason, J. A.; Chavan, S.; Crocellà, V.; Howe, J. D.; Lee, K.; Dzubak, A. L.; Queen, W. L.; Zadrozny, J. M.; Geier, S. J.; Lin, L.-C.; Gagliardi, L.; Smit, B.; Neaton, J. B.; Bordiga, S.; Brown, C. M.; Long, J. R. Reversible CO Binding Enables Tunable CO/H_2 and CO/N_2 Separations in Metal–Organic Frameworks with Exposed Divalent Metal Cations. *J. Am. Chem. Soc.* **2014**, *136*, 10752−10761.

[Ref S7] Qiu, X.-F.; Zhu, H.-L.; Huang, J.-R.; Liao, P.-Q.; Chen, X.-M. Highly Selective CO_2 Electroreduction to C_2H_4 Using a Metal–Organic Framework with Dual Active Sites. *J. Am. Chem. Soc.* **2021**, *143*, 7242–7246.

[Ref S8] Verma, S.; Ghosh, H. N. Exciton Energy and Charge Transfer in Porphyrin Aggregate/Semiconductor (TiO_2) Composites. *J. Phys. Chem. Lett.* **2012**, *3*, 1877–1884.

[Ref S9] Feng, X.; Liu, L.; Honsho, Y.; Saeki, A.; Seki, S.; Irle, S.; Dong, Y.; Nagai, A.; Jiang, D. High-Rate Charge-Carrier Transport in Porphyrin Covalent Organic Frameworks: Switching from Hole to Electron to Ambipolar Conduction. *Angew. Chem. Int. Ed.* **2012**, *51*, 2618–2622.

[Ref S10] Lin, S.; Diercks, C. S.; Zhang, Y.-B.; Kornienko, N.; Nichols, E. M.; Zhao, Y.; Paris, A. R.; Kim, D.; Yang, P.; Yaghi, O. M.; Chang, C. J. Covalent organic

frameworks comprising cobalt porphyrins for catalytic CO_2 reduction in water. *Science* **2015**, *349*, 1208–1213.

[Ref S11] Han, L.; Song, S.; Liu, M.; Yao, S.; Liang, Z.; Cheng, H.; Ren, Z.; Liu, W.; Lin, R.; Qi, G.; Liu, X.; Wu, Q.; Luo, J.; Xin, H. L. Stable and Efficient Single-Atom Zn Catalyst for CO_2 Reduction to CH_4. *J. Am. Chem. Soc.* **2020**, *142*, 12563−12567.

[Ref S12] Cheng, T.; Xiao, H.; Goddard, W. A. III. Reaction Mechanisms for the Electrochemical Reduction of CO_2 to CO and Formate on the Cu(100) Surface at 298 K from Quantum Mechanics Free Energy Calculations with Explicit Water. *J. Am. Chem. Soc.* **2016**, *138*, 13802–13805.

[Ref S13] Peterson, A. A.; Abild-Pedersen, F.; Studt, F.; Rossmeisl, J.; Nørskov, J. K. How copper catalyzes the electroreduction of carbon dioxide into hydrocarbon fuels. *Energy Environ. Sci.* **2010**, *3*, 1311–1315.
